# TET3-overexpressing macrophages promote endometriosis

**DOI:** 10.1172/JCI181839

**Published:** 2024-11-01

**Authors:** Haining Lv, Beibei Liu, Yangyang Dai, Feng Li, Stefania Bellone, Yuping Zhou, Ramanaiah Mamillapalli, Dejian Zhao, Muthukumaran Venkatachalapathy, Yali Hu, Gordon G. Carmichael, Da Li, Hugh S. Taylor, Yingqun Huang

**Affiliations:** 1 Department of Obstetrics, Gynecology and Reproductive Sciences, Yale University School of Medicine, New Haven, Connecticut, USA.; 2 Center for Reproductive Medicine and Obstetrics and Gynecology, Affiliated Hospital of Medical School, Nanjing University, Nanjing, China.; 3 Center of Reproductive Medicine, National Health Commission Key Laboratory of Advanced Reproductive Medicine and Fertility, Shengjing Hospital of China Medical University, Shenyang, China.; 4 Assisted Reproduction Unit, Department of Obstetrics and Gynecology, Sir Run Run Shaw Hospital, School of Medicine, Zhejiang University, Hangzhou, China.; 5 Department of Anesthesiology, The First Affiliated Hospital of Xi’an Jiaotong University, Xi’an, China.; 6 Yale Center for Genome Analysis, Yale University School of Medicine, New Haven, Connecticut, USA.; 7 Department of Chemistry, Yale University, New Haven, Connecticut, USA.; 8 Department of Genetics and Genome Sciences, University of Connecticut Health Center, Farmington, Connecticut, USA.; 9 Yale Center for Molecular and Systems Metabolism, Yale University School of Medicine, New Haven, Connecticut, USA.

**Keywords:** Inflammation, Reproductive biology, Macrophages, Obstetrics/gynecology, Pain

## Abstract

Endometriosis is a debilitating, chronic inflammatory disease affecting approximately 10% of reproductive-age women worldwide with no cure. While macrophages have been intrinsically linked to the pathophysiology of endometriosis, targeting them therapeutically has been extremely challenging due to their high heterogeneity and because these disease-associated macrophages (DAMs) can be either pathogenic or protective. Here, we report identification of pathogenic macrophages characterized by TET3 overexpression in human endometriosis lesions. We show that factors from the disease microenvironment upregulated TET3 expression, transforming macrophages into pathogenic DAMs. TET3 overexpression stimulated proinflammatory cytokine production via a feedback mechanism involving inhibition of let-7 miRNA expression. Remarkably, these cells relied on TET3 overexpression for survival and hence were vulnerable to TET3 knockdown. We demonstrated that Bobcat339, a synthetic cytosine derivative, triggered TET3 degradation in both human and mouse macrophages. This degradation was dependent on a von Hippel-Lindau (VHL) E3 ubiquitin ligase whose expression was also upregulated in TET3-overexpressing macrophages. Furthermore, depleting TET3-overexpressing macrophages either through myeloid-specific *Tet3* ablation or using Bobcat339 strongly inhibited endometriosis progression in mice. Our results defined TET3-overexpressing macrophages as key pathogenic contributors to and attractive therapeutic targets for endometriosis. Our findings may also be applicable to other chronic inflammatory diseases where DAMs have important roles.

## Abstract 2

We discover a specific population of disease-promoting macrophages characterized by TET3 overexpression and a potential new therapeutic agent for endometriosis, a life-impacting chronic inflammatory disease.

## Introduction

Endometriosis, defined as the growth of endometrium-like tissue outside the uterus, is a chronic inflammatory disease affecting approximately 190 million reproductive-age women worldwide (1–3). Patients suffer from years of pelvic pain, gastrointestinal symptoms, fatigue, infertility, and sometimes anxiety and depression. Endometriosis is also associated with an increased risk of epithelial ovarian cancer (4). The disease severely impacts quality of life and imposes an enormous health economic burden of roughly $69 billion annually in the US alone. Yet few treatment options are available: nonsteroidal antiinflammatory drugs (NSAIDs), hormonal contraceptives, and in refractory cases, surgery. NSAIDs work by stopping the release of prostaglandins, one of the main chemicals responsible for pain. While surgery is associated with high recurrence rates, hormone treatments have unacceptable side effects, including menopausal symptoms (2, 3, 5).

Currently, the pathophysiology of endometriosis is relatively poorly understood. Many theories of disease origin have been proposed, including those involving retrograde menstruation, hormonal, genetic, epigenetic, and immunologic factors, and endometrial stem cells (2, 3). Although there is a need for a comprehensive understanding of the etiology and pathophysiology of endometriosis, an altered immune system has emerged as a critical contributing mechanism. Macrophages, the second most abundant immune cells in endometriosis lesions after lymphocytes, play a crucial role in promoting the growth, vascularization, and innervation of the lesions as well as in producing pain symptoms (6–10).

Tissue-resident macrophages comprise both embryonic- and monocyte-derived phagocytes. These macrophages play essential roles in tissue homeostasis as well as acting as the first line of defense against pathogens. However, under pathological conditions, these macrophages can be reprogrammed by disease microenvironments to become phenotypically and functionally distinct disease-associated macrophages (DAMs), acting either to accelerate or inhibit disease progression (11). In other words, DAMs can be “pathogenic” or “protective.” DAMs function in part through secreting inflammatory mediators. The cytokines IL-1β and IL-6 are known to play important roles in the pathophysiology of endometriosis. Increased IL-1β has been found in peritoneal fluids and lesions from women with endometriosis and pelvic pain (9, 12). Notably, IL-1β promotes local neurogenesis and increases nerve density around endometriosis and endometriosis-associated pain (9). IL-6 is a pleiotropic cytokine with pro- and antiinflammatory properties depending on context (13). The proinflammatory role of IL-6 in endometriosis is supported by several lines of evidence. First, IL-6 is elevated in the peritoneal fluid and the serum of patients with endometriosis (14). Second, IL-6 released from activated macrophages stimulates the migration of endometriotic epithelial cells during formation of extrapelvic endometriosis (15). Third, treating rats with tocilizumab, an anti–IL-6 receptor monoclonal antibody, leads to substantially reduced endometriosis burden (16).

In mice, the peritoneal cavity predominantly hosts macrophages of embryonic origin, known as large peritoneal macrophages (LPMs). These LPMs are characterized by their morphology and the expression of feature markers, such as GATA6, a lineage-determining transcription factor (8, 17). LMPs can self-renew and proliferate to maintain their population constituting approximately 90% of the peritoneal macrophages (PMs) at homeostasis. The remaining 10% are smaller in size, called small peritoneal macrophages (SPMs). During inflammation and aging, these monocyte-derived macrophages (MDMs) can gradually replace embryonic-derived LPMs (8, 17). Following mobilization from bone marrow into the peritoneal cavity, these MDMs acquire most, but not all, of the phenotypical and transcriptional features of embryonic-derived LPMs (17). Additionally, there is evidence of phenotypically and functionally distinct subsets among LPMs and SPMs in the peritoneal cavity (17). In a recent study using murine models of induced endometriosis, Hogg et al. (10) elegantly demonstrated that macrophages promote or inhibit endometriosis depending on their origins. The authors described 3 origins of macrophages in mouse endometriosis lesions: eutopic endometrial tissue, infiltrating LPMs of embryonic origin, and bone marrow–derived monocytes (10). Intriguingly, after entering endometriosis lesions, these LPMs lose their signature expression of GATA6, consistent with reprogramming by the disease microenvironment (10). While reducing eutopic endometrial macrophages or substituting embryonic-derived LPMs with MDMs lessened endometriosis, inhibiting monocyte recruitment exacerbated the condition. Based on this, the authors suggested that in mice, MDMs might serve to restrain the disease, whereas macrophages from other sources could encourage its advancement (10). However, it remains to be determined whether MDMs also function to limit endometriosis in humans.

Flow cytometry has been traditionally used to study immune cells. Yet using this technique to identify specific populations of DAMs, especially those with pathogenic or protective properties, has been complicated because many populations of myeloid cells express overlapping surface/intracellular markers and macrophages change their molecular programs, surface/intracellular markers, and functional properties in a disease/disease stage–dependent manner (10, 18–21). In other words, there are no true “macrophage-specific” markers that can be used either singly or in combination in flow cytometry to accurately identify all macrophages that are pathogenic or protective without knowing which markers to start with. In addition, information about anatomical localization is essentially lost during flow cytometry sample preparation. Based on these considerations, IHC combined with single-cell RNA sequencing (scRNA-Seq) and genetic/pharmacological manipulations may be more suitable to identify these macrophages.

The TET family proteins (TET1, TET2, and TET3) oxidize 5-methylcytosine (5mC) to 5-hydroxymethylcytosine (5hmC) and its derivatives to facilitate DNA demethylation. TETs can also regulate gene expression independently of their catalytic activities (22). Altered TET expression has been shown to elicit various pathophysiological effects in a tissue/cell-dependent fashion (23–37).

Though prior studies have pinpointed macrophages as promising therapeutic targets for endometriosis, without a clear definition of molecular features specific to “pathogenic” or “protective” macrophages, development of effective therapeutic strategies would not be possible. In the current work, we used publicly available human scRNA-Seq data combined with genetic and pharmacological approaches to identify pathogenic macrophages characterized by TET3 overexpression in endometriosis. We demonstrate that these TET3-overexpressing macrophages are induced by the disease microenvironment and can be eliminated by TET3 knockdown, either via myeloid-specific *Tet3*-KO or by Bobcat339 (herein called Bc), a synthetic cytosine derivative that triggers the degradation of TET3. Importantly, this degradation relies on a specific E3 ubiquitin ligase, von Hippel-Lindau (VHL), whose expression is also upregulated in TET3-overexpressing macrophages, potentially allowing dual cell-specific therapeutic targeting.

## Results

### TET3-overexpressing macrophages are abundant in endometriosis lesions.

**Figure 1:**
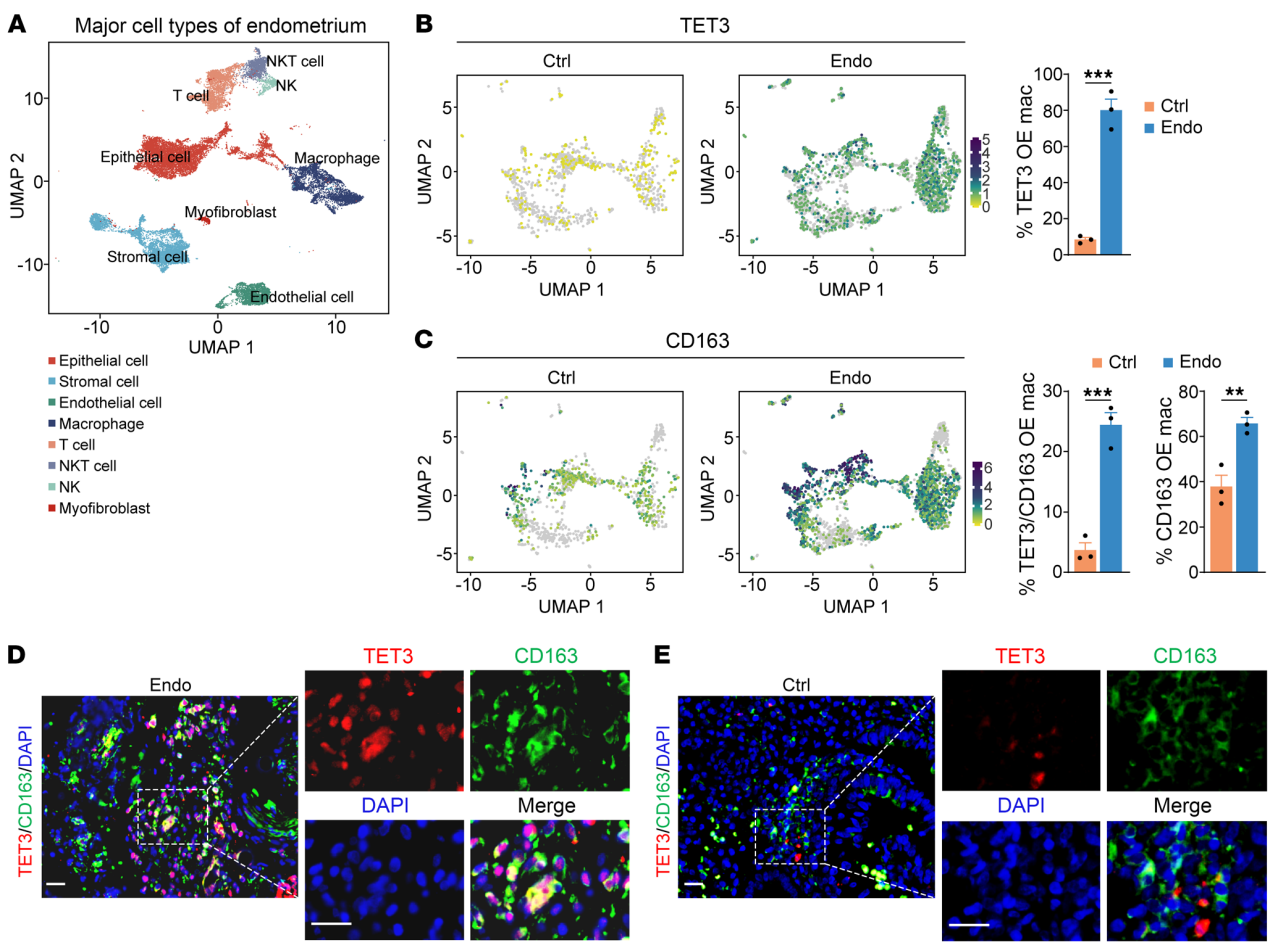
TET3 OE macrophages are abundant in human peritoneal endometriosis lesions. (**A**) UMAP plot displaying total cells from normal endometrium (Ctrl, *n* = 3) and endometriosis lesions (Endo, *n* = 3). (**B**) UMAP showing macrophage TET3 expression in Ctrl (*n* = 3) and Endo (*n* = 3), with bar graph displaying percentages of TET3 OE macrophages on the right. (**C**) UMAP showing macrophage CD163 expression in Ctrl (*n* = 3) and Endo (*n* = 3). Bar graphs on the right show percentages of CD163 OE and TET3/CD163 double-OE macrophages. Data are represented as mean ± SEM. **P < 0.01; ***P < 0.001, 2-tailed Student’s *t* test. (**D** and **E**) Representative immunofluorescence staining of TET3 (red), CD163 (green), and nuclei (blue) from human endometriosis tissue (*n* = 7) and normal endometrial tissue (*n* = 5). The panels on the right are zoomed-in images from the left. Scale bars: 40 μm.

We have previously established that chronic TET3 overexpression in human and mouse skeletal myocytes and liver hepatocytes and hepatic stellate cells is associated with insulin resistance, type 2 diabetes, and liver fibrosis (26, 36, 38, 39). We have also reported TET3 overexpression in human uterine leiomyomas (uterine fibroids) (40). Thus, chronically elevated TET3 expression appears to be intimately linked with inflammation. Supporting this notion, analysis of publicly available human scRNA-Seq datasets (6) revealed an 8-fold increase in the percentage of TET3-overexpressing macrophages (TET3 OE Mac) in peritoneal lesions from patients with endometriosis (Endo) as compared with eutopic endometrium from nonendometriosis women (Ctrl) (Figure 1, A and B). Of note, the macrophages were categorized based on multiple markers including CD163, CD68, CD14, MRC1, and CD86 (Figure 1A). The transmembrane scavenger receptor CD163 is exclusively expressed in monocytes and macrophages, with expression markedly increasing in macrophages (41, 42). Using fluorescence IHC, CD163^+^ macrophages were predominantly detected in human normal endometrium and all 3 types of endometriotic lesions (peritoneal, ovarian, and rectovaginal) (43). While CD163 high expressors (CD163 OE Mac) were abundantly detected both in Ctrl and Endo (with ~2-fold increase in Endo compared with Ctrl), there was an 8-fold increase in the percentage of TET3 OE Mac that were also CD163 high expressors (TET3/CD163 OE Mac) in Endo versus Ctrl (Figure 1C). Similar observations were made in ovarian endometriotic lesions (Supplemental Figure 1; supplemental material available online with this article; https://doi.org/10.1172/JCI181839DS1). Interestingly, the TET3 OE macrophages were predominantly detected in 2 subpopulations characterized by expression of markers often found in “tissue-resident macrophages/PMs,” though these TET3 OE macrophages did not express GATA6 (Supplemental Figure 2) (6).

Next, we analyzed macrophage TET3 protein expression in human tissue samples using fluorescence IHC. Because an 8-fold increase in the percentage of TET3 OE Mac in Endo versus Ctrl was seen using either single (CD163) or multiple (CD163, CD68, CD14, MRC1, and CD86) markers, we used CD163 as a convenient readout for macrophages. The specificity of the TET3 antibody was confirmed in TET3-knockdown human primary peripheral blood MDMs (Supplemental Figure 3). Consistent with scRNA-Seq results (Figure 1, B and C and Supplemental Figure 1), TET3 OE macrophages were abundantly detected in endometriotic lesions (Endo) (Figure 1D), but not in normal endometrium (Ctrl) (Figure 1E). In line with the high heterogeneity of endometriosis lesions within each individual and across individuals with endometriosis, TET3 OE macrophages were not uniformly distributed throughout disease tissue and varied in abundance across patients (Supplemental Figure 4). We observed heterogeneous subcellular localization of TET3 in macrophages (Figure 1, D and E, and Supplemental Figure 4). Heterogeneous subcellular localization of TET proteins has been documented in other cell types (44–46). We also noticed TET3 high expressors that were CD163 negative. These cells were present both in normal and endometriotic lesions (Figure 1, D and E, and Supplemental Figure 4) and thus did not appear to be disease specific. The identity and function of these cells remain to be investigated. Drawing from our scRNA-Seq and IHC investigations, which revealed a disease-specific correlation of TET3 OE macrophages, we postulated that these macrophages might contribute to the pathology of endometriosis.

### TET3 overexpression is induced by disease-associated factors.

**Figure 2:**
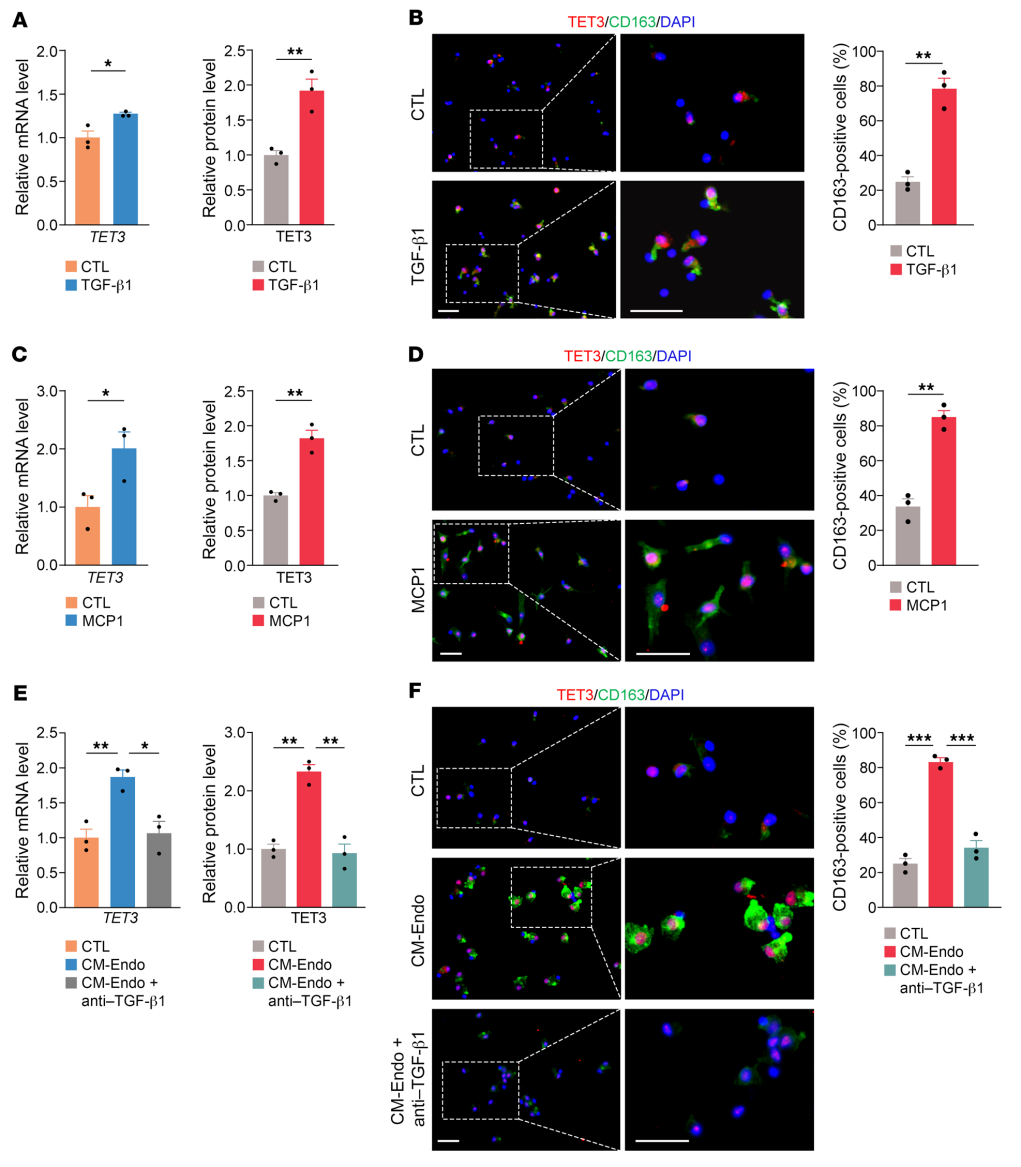
Macrophage TET3 expression is upregulated by inflammatory mediators in human MDMs. (**A**) qRT-PCR of TET3 mRNA (left) and IHC quantification of TET3 protein (right) from MDMs treated with control media (CTL) or TGF-β1 at a final concentration of 10 ng/mL for 48 hours. MFI of TET3 in **B** was used to quantify TET3 protein expression. (**B**) Representative photomicrographs and corresponding statistical analysis of immunostaining of TET3 and CD163 in MDMs treated as in **A**. (**C**) qRT-PCR of TET3 mRNA (left) and IHC quantification of TET3 protein (right) from MDMs treated with CTL or MCP1 at a final concentration of 200 ng/mL for 24 hours. MFI of TET3 (red) in **D** was used to quantify TET3 protein expression. (**D**) Representative photomicrographs and corresponding statistical analysis of immunostaining of TET3 (red) and CD163 (green) in MDMs treated as in **C**. (**E**) qRT-PCR of TET3 mRNA (left) and IHC quantification of TET3 protein (right) from MDMs treated with CTL, CM-Endo, or CM-Endo plus TGF-β1 antibody at a final concentration of 10 ng/mL for 72 hours. MFI of TET3 in **F** was used to quantify TET3 protein expression. (**F**) Representative photomicrographs and corresponding statistical analysis of immunostaining of TET3 and CD163 in MDMs treated as in **E**. For quantification of immunostaining, *n* = 3 randomly selected areas per group were used. All data are represented as mean ± SEM. *P < 0.05; **P < 0.01; ***P < 0.001, 2-tailed Student’s *t* test. Scale bars: 40 μm.

Macrophages are remarkably plastic cells that can be modified molecularly and functionally in response to microenvironmental cues (11, 47). We hypothesized that disease-associated factors might upregulate TET3 expression in macrophages. Levels of TGF-β1 were found to increase in the peritoneal cavity of women with endometriosis, and *Tgfb1* deletion reduced the growth of endometriosis lesions in mice (48). Likewise, increased secretion of MCP1 (also known as CCL2) from PMs was reported in women with versus without endometriosis (49). Also, the concentrations of MCP1 in serum and peritoneal fluid in women with endometriosis were significantly higher than in those without endometriosis (50). First, we tested TGF-β1 in macrophage colony-stimulating factor–induced (M-CSF–induced) human MDMs. TGF-β1 increased TET3 expression both at mRNA and protein levels (Figure 2A) with a concomitant increase in the number of CD163^+^ cells (Figure 2B), consistent with scRNA-Seq results (Figure 1C). Incubation of MDMs with MCP1 resulted in similar effects (Figure 2, C and D). Further, comparable results were obtained in MDMs induced by M-CSF followed by polarization with IL-4 (Supplemental Figure 5, A–C). Next, MDMs were exposed to conditioned media from human telomerase reverse transcriptase (hTERT) immortalized stromal cells derived from human endometriotic lesions (51) (CM-Endo). We observed increased TET3 expression (Figure 2E) accompanied by increased numbers of CD163^+^ cells (Figure 2F). These effects were blunted when a TGF-β1–specific antibody was used (Figure 2, E and F). In contrast, treating MDMs with conditioned media from hTERT immortalized stromal cells derived from normal human endometrium (HESC) (51) and did not increase expression of TET3 and CD163^+^ cells (Supplemental Figure 6).

At homeostasis, mouse PMs had a baseline expression of Tet3 (NCBI’s Gene Expression Omnibus [GEO] SM8001711; ref. 52) (Supplemental Figure 7). When cultured mouse PMs were examined, both TGF-β1 and MCP1 were able to increase TET3 expression and numbers of CD163^+^ cells (Supplemental Figure 8, A–D). CM-Endo elicited similar effects in a TGF-β1–dependent manner (Supplemental Figure 8, E and F). Mature human TGF-β1 shares 99% amino acid sequence identity with mouse TGF-β1, and their cross-species activity has been demonstrated (53). In aggregate, our results show that disease-associated factors can upregulate TET3 expression in macrophages.

### TET3 maintains the viability of TET3 OE macrophages.

**Figure 3:**
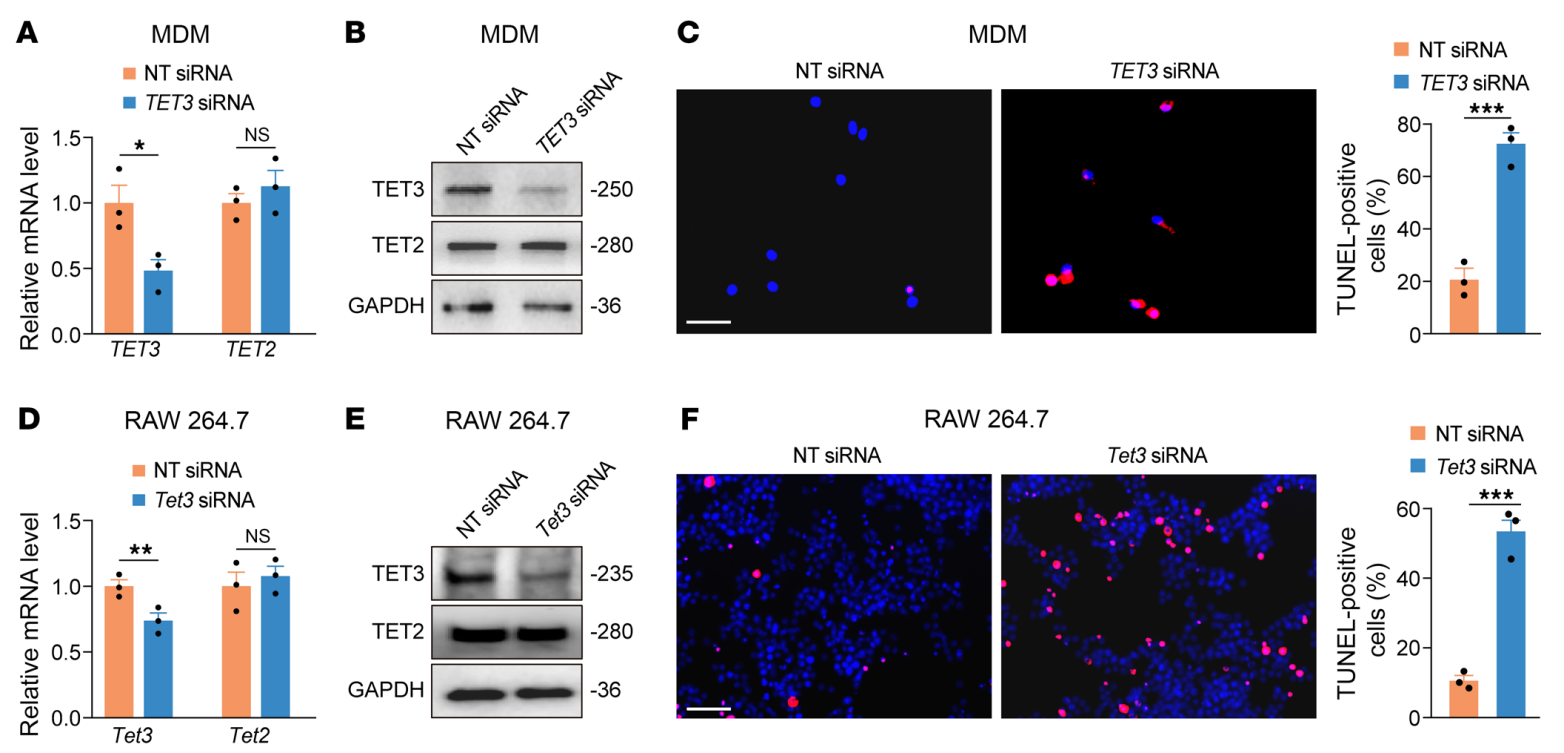
TET3 knockdown leads to apoptosis of TET3 OE macrophages. (**A**) qRT-PCR of TET3 and TET2 mRNAs isolated from MDMs treated with CM-Endo and transfected with nontargeting (NT) siRNA or *TET3* siRNA for 24 hours. *n* = 3 per group in technical replicates. (**B**) Representative immunoblots for TET3 and TET2 from MDMs treated as in **A**. Protein sizes in kDa are marked on the right. Proteins were isolated after 48 hours of transfection. (**C**) Representative photomicrographs and corresponding statistical analysis of TUNEL^+^ (red) MDMs treated as in **A**. TUNEL assays were performed after 48 hours of transfection. *n* = 3 randomly selected areas per group. (**D**) qRT-PCR of Tet3 and Tet2 mRNAs isolated from RAW 264.7 cells transfected with NT siRNA or *Tet3* siRNA for 24 hours. *n* = 3 per group in technical replicates. (**E**) Representative immunoblots for TET3 and TET2 from RAW 264.7 cells treated as in **D**. Proteins were isolated after 48 hours of transfection. (**F**) Representative photomicrographs and corresponding statistical analysis of TUNEL^+^ RAW 264.7 cells treated as in **D**. TUNEL assays were performed after 48 hours of transfection. *n* = 3 randomly selected areas per group. *P < 0.05; **P < 0.01; ***P < 0.001, 2-tailed Student’s *t* test. Scale bars: 40 μm.

To explore the functional significance of TET3 overexpression, we downregulated TET3 using an siRNA specifically targeting human *TET3* (*TET3* siRNA) (31) in M-CSF–induced MDMs exposed to CM-Endo shown to induce TET3 overexpression (Figure 2, E and F). Transfection of *TET3* siRNA resulted in decreased expression of TET3 mRNA (Figure 3A) and protein (Figure 3B) without affecting TET2 expression (Figure 3, A and B). TET3 and TET2 are the predominant TET family isoforms expressed in macrophages (54, 55). Importantly, TET3 knockdown induced apoptosis, as assessed by the TUNEL assay (Figure 3C). Similar results were obtained in M-CSF/IL-4–induced MDMs (Supplemental Figure 5, D and E). Likewise, TET3 knockdown using an siRNA specifically targeting mouse *Tet3* (*Tet3* siRNA) (31) in unstimulated mouse RAW 264.7 macrophages increased apoptosis (Figure 3, D–F). Raw 264.7 macrophages intrinsically overexpress TET3 without stimulation (Supplemental Figure 9). These results suggest that maintaining a high level of TET3 expression is required for preventing apoptosis in TET3 OE macrophages.

### Bc destabilizes TET3 via recruiting the VHL E3 ubiquitin ligase.

The catalytic domains of the 3 TET enzymes are highly conserved, though each of the members exhibits distinct catalytic activity and substrate preferences (56). Bc, a synthetic cytosine derivative, was predicted to bind specifically to the catalytic domains of all 3 TET proteins and inhibits their catalytic activity (57, 58). However, Bc elicited its catalytic inhibitory activity in vitro only in the presence of 125 μM of copper(II) (58). We have recently documented that Bc destabilizes TET3 protein in cultured neuronal cells with or without copper(II), albeit the underlying mechanism was not defined (59). It is not unprecedented that small molecules initially designed as protein function modulators are later serendipitously found to induce protein degradation (60, 61).

**Figure 4:**
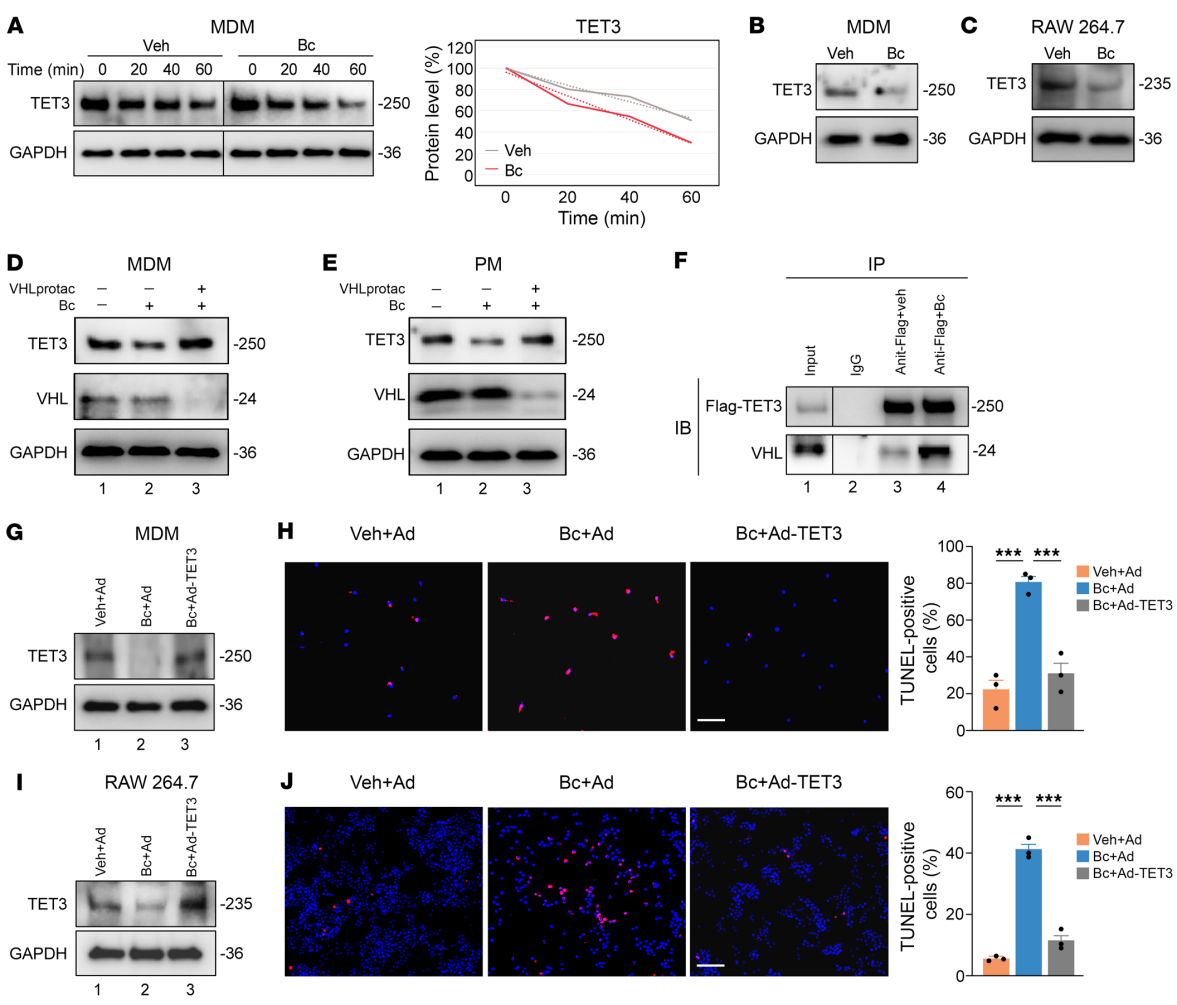
Bc induces apoptosis of TET3 OE macrophages. (**A**) Human MDMs were incubated with Veh or Bc at a final concentration of 10 μM for 2 hours, followed by time-course analysis of TET3 in the presence of CHX at a final concentration of 50 μg/mL. Cells were harvested at 0, 20, 40, and 60 minutes after addition of CHX. Human MDMs in CM-Endo (**B**) and RAW 264.7 cells (**C**) were incubated with Veh or Bc at a final concentration of 10 μM for 24 hours. Proteins were extracted and analyzed. (**D**) Immunoblots for TET3 and VHL in MDMs pretreated with or without VHLprotac at the concentration of 5 μM for 18 hours, followed by exposure to Bc at 10 μM for 8 hours. (**E**) PMs from WT mice primed with 30 ng/mL of TGF-β1 were conditioned by VHLprotac 5 μM for 18 hours, followed by exposure to Bc at 10 μM for 8 hours and Western blot analysis. (**F**) co-IP of Flag-TET3 and endogenous VHL in H1299 cells transfected with Ad-TET3, with or without the presence of Bc at 50 μM for 2 hours. MDMs (**G** and **H**) (primed with 10 ng/mL of TGF-β1) and RAW 264.7 cells (**I** and **J**) were incubated with Veh plus GFP-expressing adenovirus (Veh+Ad), Bc at 10 μM plus Ad (Bc+Ad), or Bc at 10 μM plus TET3-expressing adenovirus (Bc+Ad-TET3) for 48 hours, followed by immunoblotting and TUNEL assays. Representative immunoblots, photomicrographs and corresponding statistical analysis are shown. For TUNEL assay, *n* = 3 randomly selected areas per group. All data are represented as mean ± SEM. ***P < 0.001, 1-way ANOVA with Tukey’s post test (**H** and **J**). Scale bars: 40 μm. The dashed dividing lines (**A** and **F**) indicate splicing of noncontiguous lanes from the same blots.

To determine whether Bc destabilizes TET3 also in macrophages, we performed time-course assays on MDMs in the presence of cycloheximide (CHX), a protein synthesis inhibitor. The half-life of TET3 was approximately 40 minutes and approximately 70 minutes in the presence and absence of Bc, respectively (Figure 4A). This was consistent with the observation that after Bc treatment, the steady-state level of TET3 was markedly reduced both in human MDMs (Figure 4B) and mouse RAW 264.7 cells (Figure 4C).

The VHL protein is the substrate recognition subunit of an E3 ubiquitin ligase shown to target TET proteins (substrates) for ubiquitination and subsequent proteasomal degradation (62). To investigate whether the destabilization of TET3 induced by Bc might be mediated through VHL, we examined the effect of VHL knockdown using a PROTAC-based small molecule VHL degrader (Homo-PROTAC pVHL30 degrader 1, herein called VHLprotac) (63). Human MDMs were pretreated without or with VHLprotac for 18 hours (to lower endogenous VHL), followed by Bc treatment for 8 hours. While Bc expectedly decreased TET3 protein levels (Figure 4D), it failed to do so when VHL was downregulated. Similar observations were made when mouse primary PMs were examined (Figure 4E). These results suggest that VHL is required for Bc-induced TET3 destabilization. Using coimmunoprecipitation (co-IP) assays, TET3 was shown to form protein complexes with VHL in H1299 cells (a human lung cancer cell line) where VHL overexpression accelerated TET3 degradation (62). We hypothesized that Bc might facilitate the complex formation between TET3 and VHL, thereby promoting TET3 degradation. To test this, we performed co-IP experiments using H1299 cells that were expressing a Flag-tagged human TET3 via an adenoviral vector (Ad-TET3) (59). While the Flag antibody pulled down comparable amounts of Flag-TET3 (Figure 4F), more VHL was detected in Flag-TET3–containing protein complexes in cells treated with Bc. These results imply that Bc recruits VHL and thereby promotes the degradation of TET3.

### Bc induces apoptosis of TET3 OE macrophages.

Given that siRNA-mediated TET3 knockdown induces apoptosis of TET3 OE macrophages (Figure 3, C and F) and that Bc destabilizes TET3 protein (Figure 4, A–C), we explored whether exposing TET3 OE macrophages to Bc would promote apoptosis, and if such an effect could be counteracted by introducing exogenous TET3. Therefore, human MDMs were treated with Bc in the presence or absence of exogenous TET3 expression from Ad-TET3. While Bc decreased the level of TET3 protein, exogenous TET3 expression reinstated it to the baseline level (Figure 4G). Exposure of these cells to Bc induced apoptosis, a response that was nullified upon restoration of TET3 to control levels through exogenous expression (Figure 4H). Similar observations were made in RAW 246.7 cells (Figure 4, I and J). Thus, Bc promotes apoptosis of TET3 OE macrophages in a TET3-dependent manner. Of note, it is extremely unlikely that the cell death induced by Bc was due to an inhibition of the catalytic activity of TET proteins. The intracellular concentrations of free Cu ions are in the range of 10^–15^ to 10^–21^ M (64), whereas a concentration of 125 μM of Cu^2+^ was necessary for Bc to inhibit the catalytic activity of TET proteins (58). Though the possibility that Bc may induce degradation of other proteins cannot be excluded, our data suggest that Bc-induced TET3 degradation is a major contributor to the decreased viability of TET3 OE macrophages.

### TET3 affects genome-wide gene expression in macrophages.

We performed siRNA knockdown of TET3 in RAW 264.7 cells, followed by RNA deep-sequencing (RNA-Seq). TET3 knockdown led to altered expression of 342 genes (*P* < 0.05) when compared with control siRNA-transfected cells (Supplemental Figure 10A). Ingenuity Pathway Analysis (IPA) identified cytokine/chemokine, pattern recognition receptor, and cell death signaling to be among the top classes affected by TET3 (Supplemental Figure 10B). In TET3-knockdown cells, genes in the cell death signaling class with known proapoptosis functions were mostly upregulated and genes in the cytokine/chemokine class with known proinflammatory actions were mostly downregulated (Supplemental Figure 10C). *Bcl2l11* (encoding BIM), *Bid* (encoding BID), and *Pmaip1* (encoding NOXA) are key proapoptotic genes of the *Bcl-2* family (65). Consistent with TET3 knockdown inducing apoptosis (Figure 3, A–F), quantitative reverse-transcriptase PCR (qRT-PCR) analysis revealed significantly increased expression of *Bcl2l11*, *Bid*, and *Pmaip1* in RAW 264.7 macrophages as well as in TGF-β1 primed human MDMs transfected with TET3 siRNAs versus control siRNAs (Supplemental Figure 10, D and E). When TGF-β1–primed mouse PMs were tested, we observed upregulation of all 3 proapoptosis genes in Bc- versus Veh-treated cells (Supplemental Figure 10F), consistent with Bc-induced apoptosis of macrophages (Supplemental Figure 10, G and H, and Figure 4, G–J). These results suggest that TET3 protects TET3 OE macrophages from apoptosis at least in part through inhibition of expression of key proapoptosis genes, though other pathways are likely also involved.

### TET3 positively regulates proinflammatory gene expression.

The lysozyme gene *Lyz2* which encodes lysozyme M (LysM) is exclusively expressed in myelomonocytic cells including monocytes, macrophages, and granulocytes (66). We mated *Tet3^fl/fl^* mice with *LysM-cre* mice to KO *Tet3* in the myelomonocytic compartment (*Lysm*^+/WT^ *Tet3^fl/fl^*, herein referred to as Mye-Tet3–KO; *Lysm*^WT/WT^ *Tet3^fl/fl^*, herein WT). At homeostasis, PMs from Mye-Tet3–KO mice showed approximately 90% decrease in the expression of *Tet3* without affecting *Tet2* (Supplemental Figure 11A), consistent with the notion that approximately 90% of PMs are LysM^+^ (66). In TET3 OE macrophages, knocking down TET3 led to apoptosis (Figure 3). One might expect this to affect the numbers and percentages of myeloid populations due to the loss of *Tet3*. However, this was not observed. Mye-Tet3–KO mice, compared with WT, showed similar myeloid population counts and ratios, including macrophage and monocytic cells in spleen and bone marrow (Supplemental Figure 12). This suggests that *Tet3* ablation did not alter steady-state myeloid cell lineage distribution. Importantly, there was no significant change in the numbers of PMs between WT and Mye-Tet3–KO mice (Supplemental Figure 11B). This outcome was not surprising because, first, TET3 expression is not essential for monocyte development and differentiation (67, 68). Second, under pathological conditions, macrophage TET3 overexpression is induced (Figure 1, Figure 2, and Supplemental Figures 4 and 8). Once overexpressed, these macrophages transform into a transcriptionally and functionally distinct population (see below) and become susceptible to TET3 knockdown (Figure 3 and Figure 4, G–J). During homeostasis, macrophages do not exhibit TET3 overexpression and therefore are not sensitive to TET3 knockdown. Further, myeloid-specific *Tet3* ablation did not alter body weight, body composition, or fasting glucose levels (Supplemental Figure 13).

**Figure 5:**
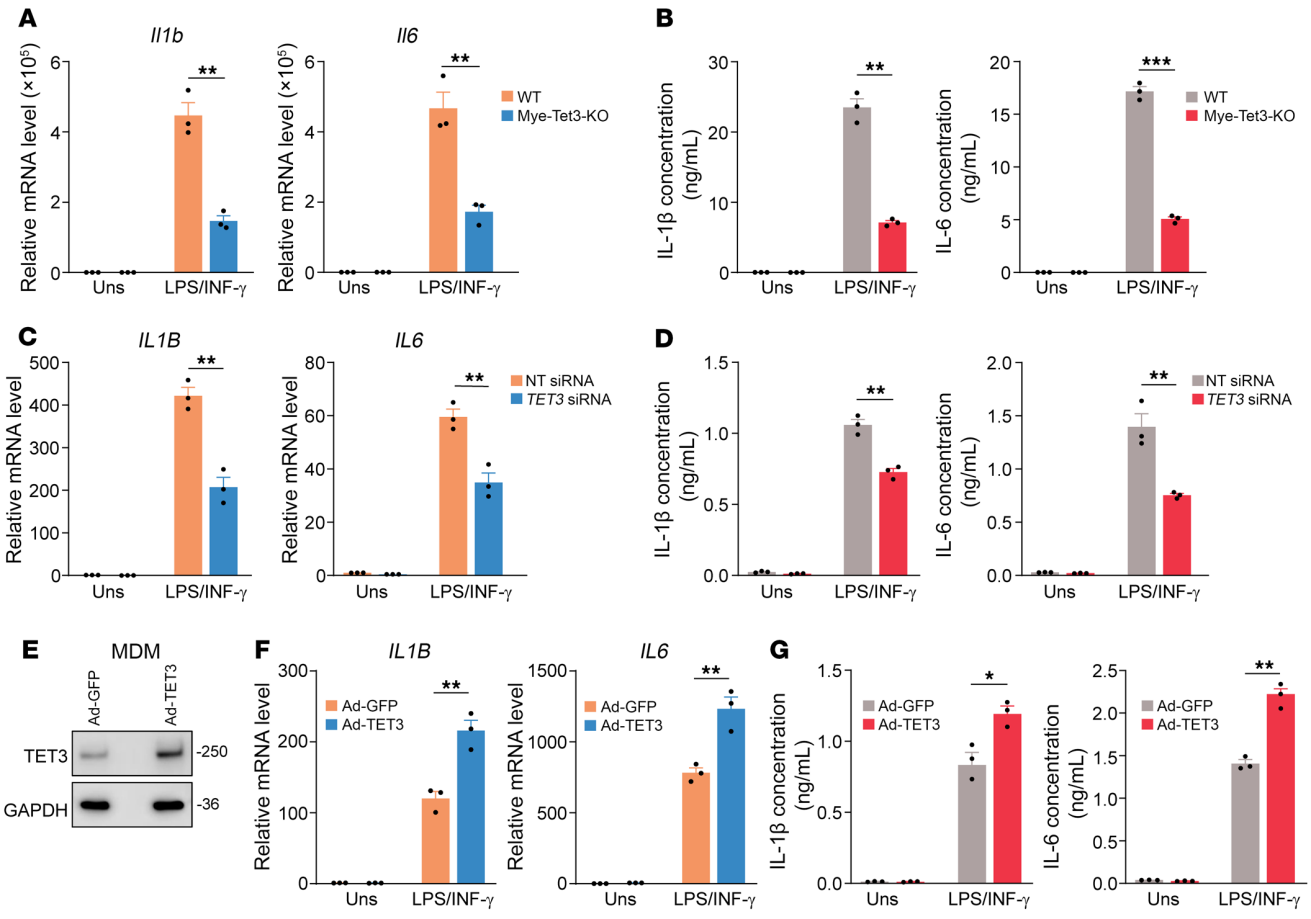
TET3 knockdown reduces macrophage expression of IL-1β and IL-6. (**A**) qRT-PCR of Il1b and Il6 mRNAs of cultured PMs isolated from Mye-Tet3–KO mice or WT controls and treated with 10 ng/mL LPS plus 20 ng/mL IFN-γ. RNAs were isolated after 6 hours of LPS/IFN-γ stimulation. Uns, unstimulated. *n* = 3 mice per genotype. (**B**) ELISA analysis (after 6 hours of LPS/IFN-γ stimulation) of IL-1β and IL-6 of cultured PMs treated as in **A**. *n* = 3 mice per genotype. (**C**) Human MDMs primed with 10 ng/mL of TGF-β1 were transfected with NT siRNA or *TET3* siRNA. After 48 hours of transfection, cells were stimulated with 10 ng/mL LPS plus 20 ng/mL IFN-γ for 8 hours, followed by RNA extraction and qRT-PCR of IL-1B and IL-6 mRNAs. *n* = 3 biological replicates. (**D**) ELISA analysis (after 8 hours of LPS/IFN-γ stimulation) of IL-1β and IL-6 of MDMs following treatment as in **C**. (**E**) Human MDMs were infected with Ad-GFP or Ad-TET3. The next day, proteins were extracted, followed by Western blot analysis. The Ad-TET3–infected cells showed approximately 5-fold TET3 overexpression as compared with Ad-GFP–infected cells. (**F**) Human MDMs were infected with Ad-GFP or Ad-TET3. The next day, cells were stimulated with 10 ng/mL LPS plus 20 ng/mL IFN-γ for 8 hours, followed by RNA extraction and qRT-PCR of IL-1B and IL-6 mRNAs. *n* = 3 biological replicates. (**G**) ELISA analysis (after 8 hours of LPS/IFN-γ stimulation) of IL-1β and IL-6 of MDMs following treatment as in **F**. All data are represented as mean ± SEM. *P < 0.05; **P < 0.01; ***P < 0.001, 2-tailed Student’s *t* test.

Next, we examined expression of proinflammatory genes in PMs isolated from WT and Mye-Tet3–KO mice and stimulated with a combination of LPS and IFN-γ. qRT-PCR analysis showed markedly decreased expression of proinflammatory cytokines (Figure 5A and Supplemental Figure 14A), chemokines (Supplemental Figure 14B), and enzymes (Supplemental Figure 14C) in PMs isolated from Mye-Tet3–KO mice as compared with WT mice. TET3 deficiency also led to decreased production of IL-1β and IL-6 proteins (Figure 5B). Significantly decreased expression of IL-1β and IL-6 were also observed in TET3 knockdown, M-CSF–induced MDMs (Figure 5, C and D), whereas TET3 overexpression led to opposite effects both in M-CSF–induced (Figure 5, E–G) and M-CSF/IL-4–induced (Supplemental Figure 5, F and G) MDMs. Overall, our data demonstrate that TET3 upregulates the expression of proinflammatory genes.

### Myeloid-specific Tet3-KO mitigates endometriosis in mice.

**Figure 6:**
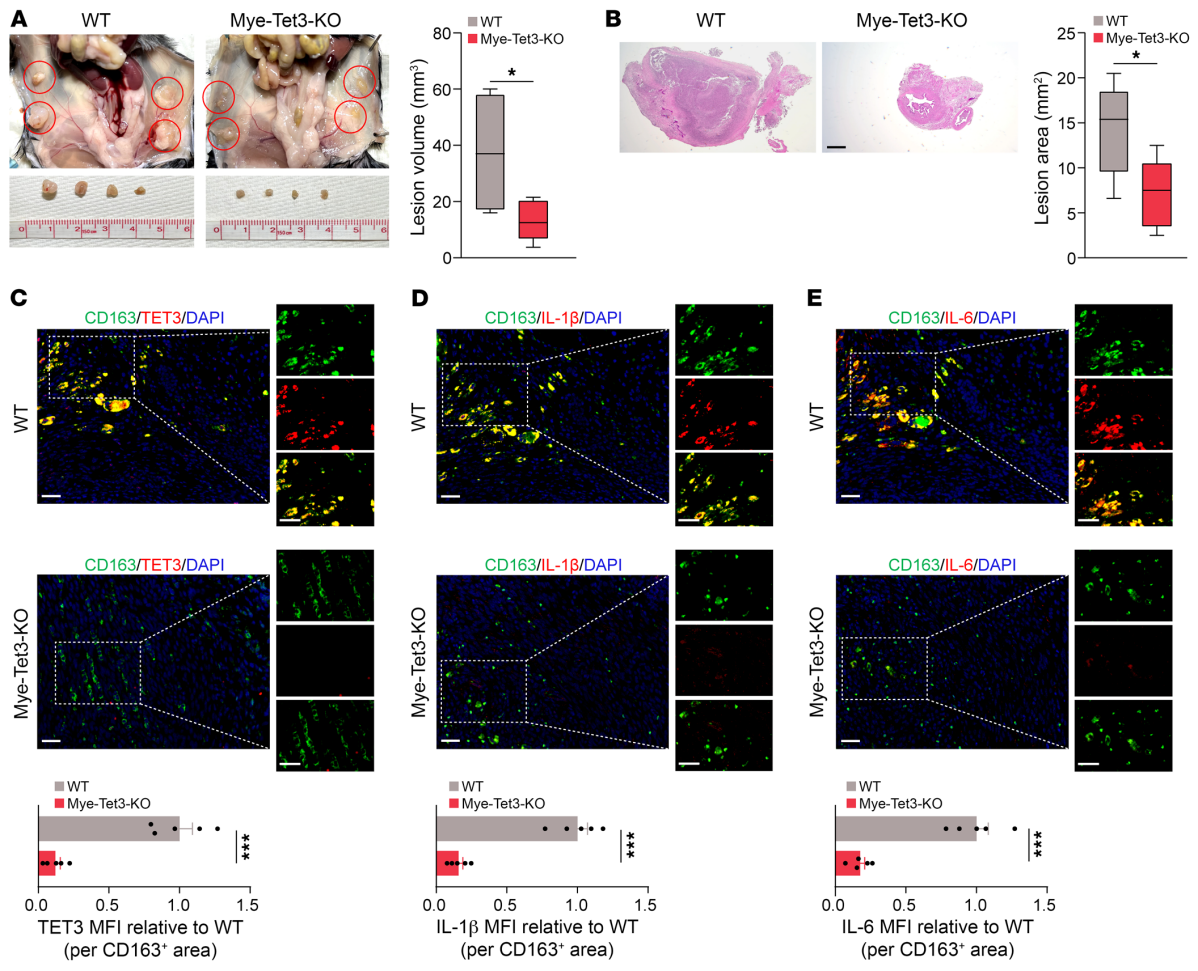
Myeloid-specific TET3-KO decreases endometriosis burden. (**A**) Representative photographs and corresponding statistical analysis of endometriosis lesions (marked by red circles). *n* = 5 mice per genotype. (**B**) Representative photomicrographs and corresponding statistical analysis of endometriosis lesions stained with H&E. *n* = 5 mice per genotype. Scale bar: 500 μm. (**C**) Immunostaining of TET3 (red) in CD163^+^ macrophages (green) and quantification of macrophage TET3 MFI in endometriosis lesions. *n* = 5 mice per genotype. (**D**) Immunostaining of IL-1β (red) and CD163^+^ macrophages (green) and quantification of macrophage IL-1β MFI in endometriosis lesions. *n* = 5 mice per genotype. (**E**) Immunostaining of IL-6 (red) and CD163^+^ macrophages (green) and quantification of macrophage IL-6 MFI in endometriosis lesions. *n* = 5 mice per genotype. *P < 0.05; ***P < 0.001. Scale bars: 40 μm (**C**–**E**).

Since disease microenvironments can induce TET3 overexpression in macrophages (Figure 1, Figure 2, and Supplemental Figures 4 and 8) and TET3 positively regulates proinflammatory gene expression (Figure 5 and Supplemental Figure 14), we hypothesized that eliminating TET3 OE macrophages may provide therapeutic effects. First, we used a surgically induced murine model of endometriosis (8, 69). Female mice of WT and Mye-Tet3–KO were subjected to surgery (week 0) to induce i.p. endometriosis, followed by euthanasia and blood and tissue collection on week 6. While no body weight difference between the groups was observed (Supplemental Figure 15), the KO mice showed a significant reduction in lesion volume both macroscopically (Figure 6A) and histologically (Figure 6B). IHC analysis revealed a marked decrease in the number of TET3 OE macrophages (Figure 6C), which was accompanied by a dramatic reduction in the expression of IL-1β (Figure 6D) and IL-6 (Figure 6E) in the lesions of KO mice as compared with WT mice. Thus, *Tet3* ablation in the myeloid compartment substantially reduces lesional TET3 OE macrophages, proinflammatory cytokine production, and endometriosis burden.

### Bc mimics the therapeutic effects of myeloid-specific Tet3-KO.

**Figure 7:**
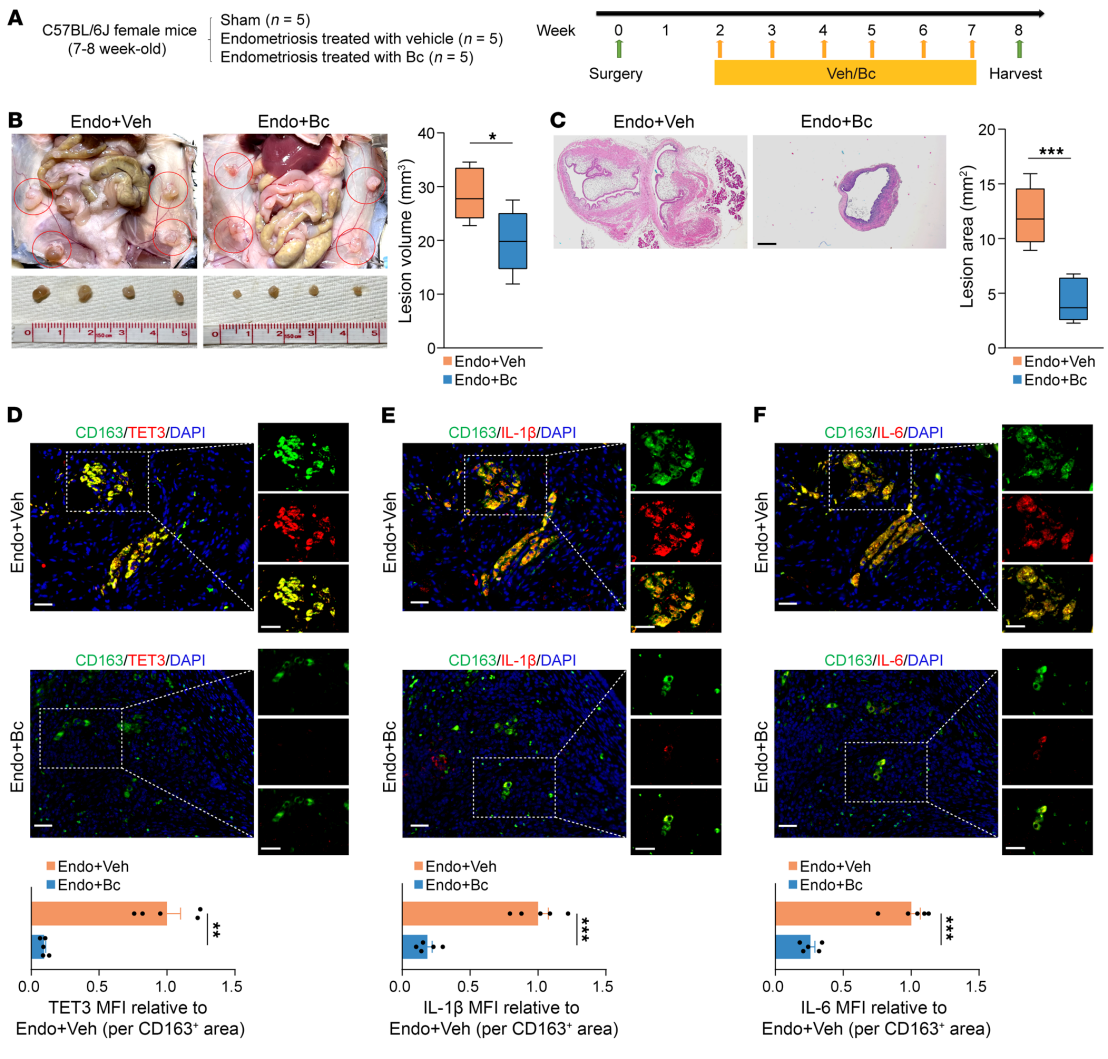
Bc recapitulates the therapeutic effects of myeloid-specific TET3-KO. (**A**) Experimental design. (**B**) Representative photographs and corresponding statistical analysis of endometriosis lesions (marked by red circles). *n* = 5 mice per group. (**C**) Representative photomicrographs and corresponding statistical analysis of endometriosis lesions stained with H&E. *n* = 5 mice per group. Scale bar: 500 μm. (**D**) Immunostaining of TET3 (red) in CD163^+^ macrophages (green) and quantification of macrophage TET3 MFI in endometriosis lesions. *n* = 5 mice per group treated as indicated. (**E**) Immunostaining of IL-1β (red) and CD163^+^ macrophages (green) and quantification of macrophage IL-1β MFI in endometriosis lesions. *n* = 5 mice per group treated as indicated. (**F**) Immunostaining of IL-6 (red) and CD163^+^ macrophage (green) and quantification of macrophage IL-6 MFI in endometriosis lesions. *n* = 5 mice per group treated as indicated. All data are represented as mean ± SEM. *P < 0.05; **P < 0.01; ***P < 0.001, 2-tailed Student’s *t* test. Scale bar: 40 μm (**D**–**F**).

WT female mice were randomly divided into 3 groups (sham, endometriosis treated with vehicle [Veh]), and endometriosis treated with Bc) and subjected to surgery (week 0) to induce endometriosis or sham (Figure 7A). The first once-a-week i.p. injection of Bc (or Veh) at a dose of 3 mg/kg body weight was performed on week 2, followed by euthanasia and sample collection on week 8. Bc treatment significantly reduced the lesion volume both macroscopically and histologically (Figure 7, B and C). Importantly, there was a marked decrease in the number of TET3 OE macrophages (Figure 7D) paralleled by a dramatic reduction in the expression of IL-1β (Figure 7E) and IL-6 (Figure 7F) in the lesions of Bc- versus Veh-treated mice. There was no evidence of liver toxicity following 6 weeks of Bc treatment (Supplemental Figure 16A), nor was there a significant difference in body weight between the Endo+Veh and Endo+Bc groups (Supplemental Figure 16B). Further, Bc treatment did not affect fertility, based on the litter size and grossly normal appearance of the newborns (Supplemental Figure 17). Collectively, these results show that TET3 OE macrophages are pathogenic and Bc can therapeutically eliminate them.

### TET3 enhances IL-1β and IL-6 expression by decreasing let-7 miRNA levels.

**Figure 8:**
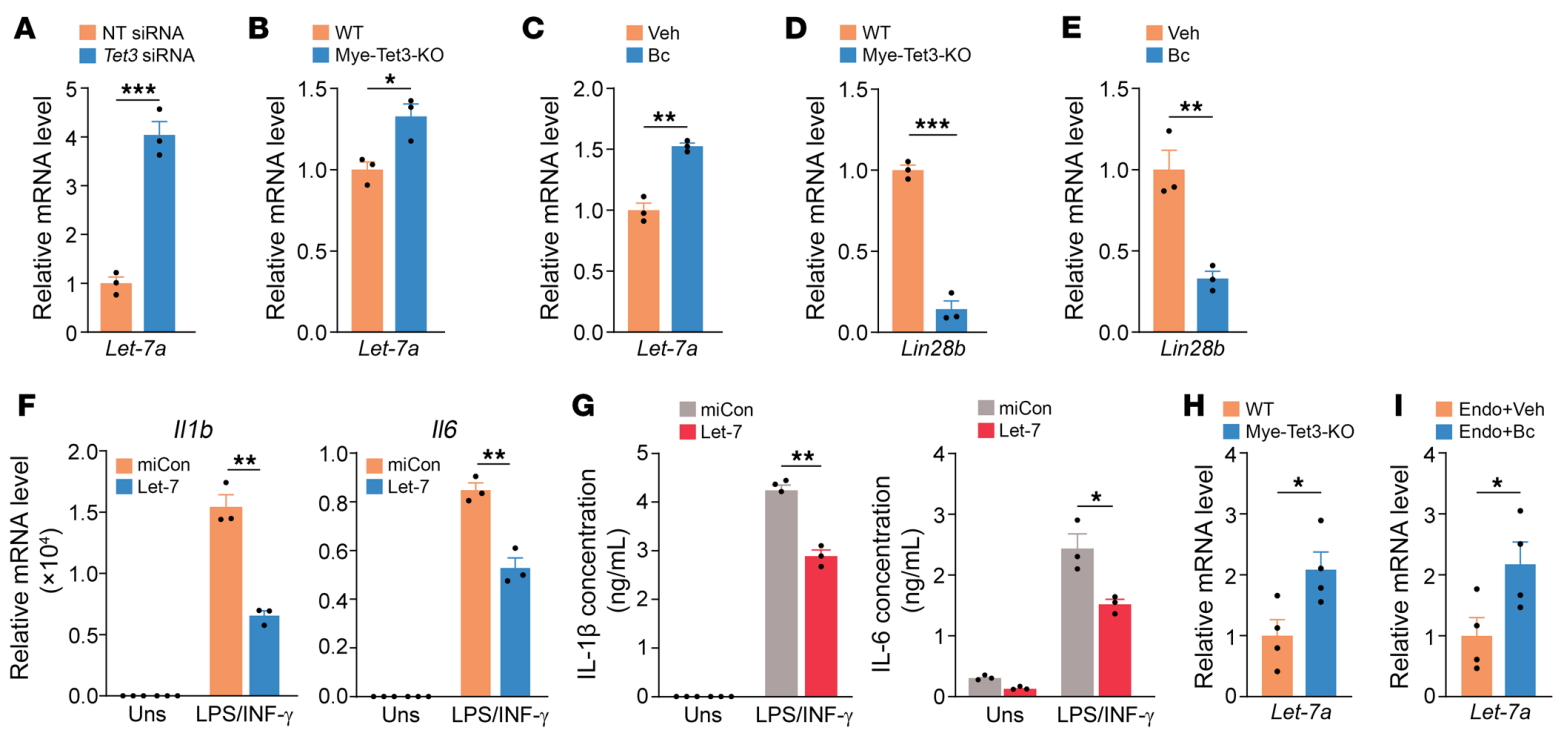
TET3 enhances IL-1β and IL-6 expression by decreasing let-7 miRNA levels. (**A**) qRT-PCR of let-7a in RAW 264.7 cells transfected with NT siRNA or *Tet3* siRNA. RNA was isolated at 24 hours after transfection. *n* = 3 technical replicates. (**B**) qRT-PCR of let-7a in PMs isolated from WT and Mye-Tet3–KO mice. *n* = 3 mice per genotype. (**C**) PMs were isolated from WT mice and treated with TGF-β1 at a final concentration of 30 ng/mL. After 48 hours, Veh or Bc was added at a final concentration of 10 μM and incubation carried out for 48 hours. RNAs were extracted and analyzed by qRT-PCR. *n* = 3 mice per group. (**D**) qRT-PCR of Lin28b mRNA in PMs isolated from WT and Mye-Tet3–KO mice. *n* = 3 mice per genotype. (**E**) qRT-PCR of Lin28b mRNA in PMs isolated from WT mice and treated as in **C**. *n* = 3 mice per group. (**F**) qRT-PCR of Il-1b and Il-6 mRNAs of PMs isolated from WT mice and transfected with control miRNA (miCon) or let-7a mimic and stimulated with 10 ng/mL LPS plus 20 ng/mL IFN-γ. RNAs were isolated after 6 hours of LPS/IFN-γ stimulation. *n* = 3 mice per group. (**G**) ELISA results of IL-1β (after 6 hours of LPS/IFN-γ stimulation) and IL-6 (after 10 hours of LPS/IFN-γ stimulation) of PMs treated as in **F**. *n* = 3 mice per group. (**H**) Relative let-7a miRNA levels in endometriosis lesions from WT and Mye-tet3–KO mice. *n* = 4 animals per genotype. (**I**) Relative let-7a miRNA levels in endometriosis lesions from Veh- or Bc-treated mice. *n* = 4 animals per group. All data are represented as mean ± SEM. *P < 0.05; **P < 0.01; ***P < 0.001, 2-tailed Student’s *t* test.

The transcription factor NF-κB induces expression of proinflammatory cytokines including IL-1β and IL-6, both of which also activate NF-κB, forming a positive feedback loop (70). Let-7 miRNAs posttranscriptionally suppress IL-6 expression by directly targeting the 3′ UTR of its mRNA, thereby indirectly inhibiting NF-κB signaling (71). The let-7 family contains 12 members that are made as precursors and are then processed to become mature miRNAs (72). Notably, let-7 miRNA levels were significantly reduced in human endometriosis tissue (73, 74). In addition, i.p. injection of let-7 decreased endometriosis lesion size (69). We hypothesized that TET3 might affect let-7 expression in macrophages. Because of the redundancy between let-7 family members, we tested let-7a. Let-7a levels increased in RAW 264.7 macrophages following treatment with *Tet3* siRNA (Figure 8A). Likewise, let-7a levels were higher in Tet3-KO macrophages than in WT macrophages (Figure 8B). Further, in Bc-treated macrophages where TET3 protein was shown to be downregulated (Supplemental Figure 10G), we detected higher levels of let-7a as compared with Veh-treated macrophages (Figure 8C). Taken together, these results suggest that TET3 negatively regulates let-7 miRNA expression. Further, the expression of let-7 miRNAs is regulated by LIN28B, which selectively blocks let-7 biogenesis through repression of let-7 processing (75). Consistent with this mechanism, LIN28B expression was decreased in Tet3-KO versus WT macrophages (Figure 8D) as well as in Bc- versus Veh-treated macrophages (Figure 8E). These results suggest that TET3 reduces let-7 miRNA levels by upregulating LIN28B. Next, we transfected WT mouse macrophages with let-7a (or control miRNA), followed by stimulation with LPS/IFN-γ. We observed a significant decrease in expression of IL-1β and IL-6 both at mRNA (Figure 8F) and protein levels (Figure 8G) in let-7a versus control transfected macrophages. Finally, let-7a miRNA levels were increased by Tet3-KO (Figure 8H) or Bc treatment (Figure 8I) in mouse endometriosis tissues. Altogether, our results suggest a mechanism by which TET3 overexpression in macrophages promotes IL-1β and IL-6 expression in part through modulating let-7 miRNA levels.

## Discussion

**Figure 9:**
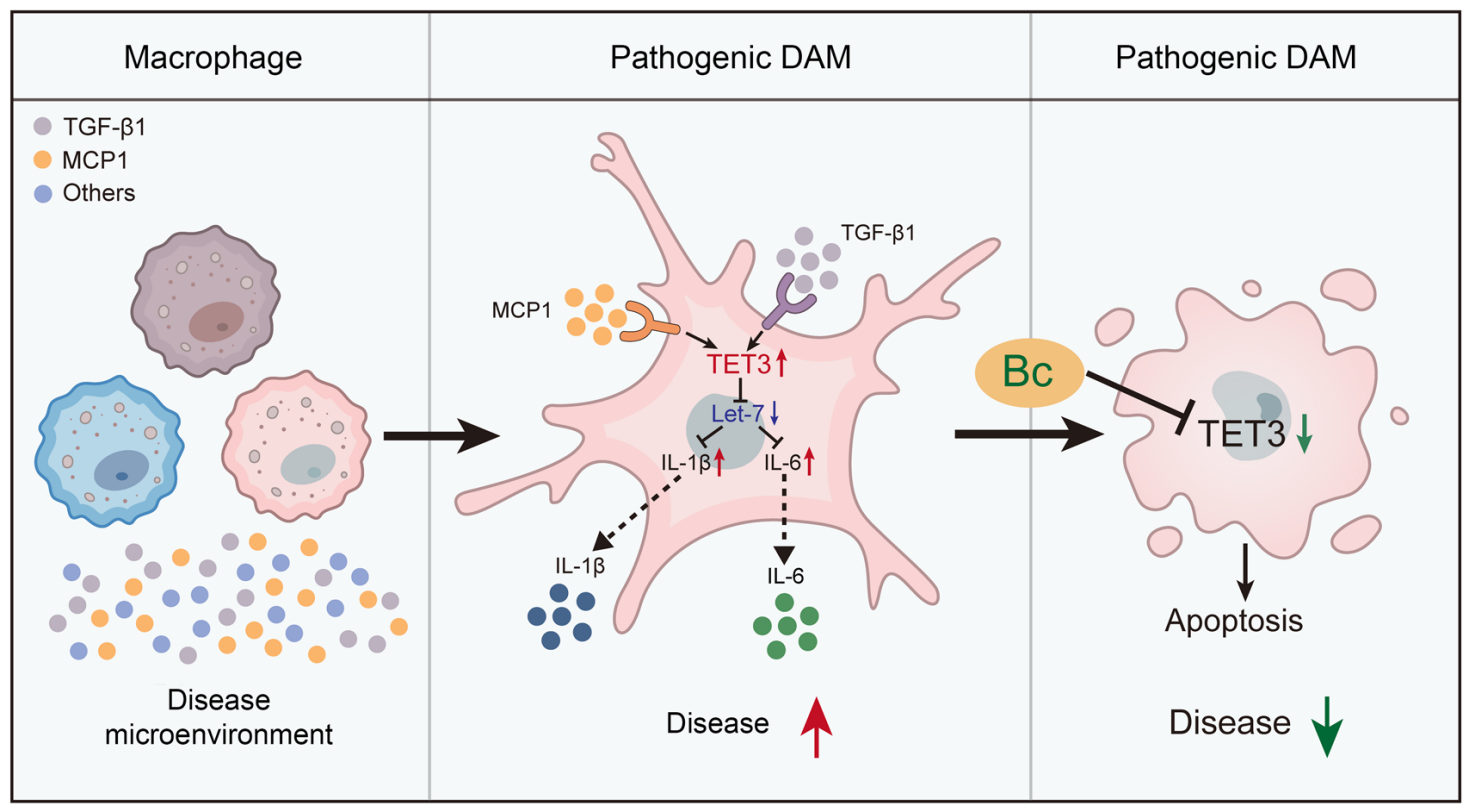
A proposed model. Macrophages exist as a heterogeneous population. Factors from the disease microenvironment induce TET3 overexpression in a subset of them. TET3 overexpression leads to genome-wide gene expression changes, which transform these macrophages into pathogenic DAMs. Though these macrophages are likely not identical in terms of other molecular features/surface markers, they share the feature of being proinflammatory and addicted to TET3 overexpression for survival. TET3 stimulates IL-1β and IL-6 production by inhibiting let-7 miRNA expression. Bc induces TET3 degradation, thereby eradicating TET3-overexpressing DAMs and inhibiting disease progression.

In this study, we describe the identification of TET3 OE macrophages in endometriosis. We present evidence demonstrating that TET3 overexpression in these cells is induced by the disease microenvironment. This upregulation of TET3 alters gene expression genome wide, transforming these cells into pathogenic DAMs. TET3 acts as a positive regulator for the expression of proinflammatory genes, such as IL-6 and IL-1β, both of which play well-documented roles in the pathology of endometriosis. From a mechanistic standpoint, TET3 suppresses the expression of let-7 miRNAs, which are known to downregulate IL-6 expression and thereby reduce NF-κB activity. Additionally, we demonstrate that TET3 increases the expression of LIN28B, which has been previously shown to specifically block the processing and accumulation of let-7 miRNAs. Owing to its activity as a DNA demethylase, it is likely that LIN28B expression is stimulated by TET3 at an epigenetic level, a possibility that warrants future investigation. Another key discovery is that overexpression of TET3 is essential for inhibiting apoptosis in TET3 OE macrophages, making them susceptible to TET3 degradation. In light of these insights, we present a proposed working model illustrated in Figure 9.

Our findings reveal that TET3 in macrophages acts as a positive regulator of inflammation, as evidenced by our experiments conducted both in vitro and in vivo. This starkly contrasts with reports indicating that macrophage TET2 functions in a divergent manner, acting to restrain expression of proinflammatory cytokines and chemokines (23–25, 28, 33–35), highlighting nonoverlapping functions of TET family proteins. Interestingly, and perhaps importantly, there was an approximately 25% increase in Tet3 mRNA expression in *Tet2*^–/–^ compared with WT macrophages, as reported (GEO GSE223694; ref. 33). Therefore, a potential role of TET3 in the enhanced inflammation observed in these *Tet2*^–/–^ mice cannot be ruled out. Moreover, an in vitro study reported that macrophage TET3 inhibits *Infb* gene transcription (54), a finding that is inconsistent with the data we have presented here. Yet it remains unclear whether this regulatory effect also takes place in vivo.

TET proteins initiate DNA demethylation by converting 5mC to 5hmC, which are further oxidized into 5-formylcytosines (5fC) and 5-carboxylcytosines (5caC), which are removed by thymine DNA glycosylase, completing the cytosine demethylation cycle (22, 76, 77). 5hmC also acts as a stable epigenetic mark and functions to enhance or inhibit binding of regulatory protein factors in a context-dependent manner (78, 79). Further, TET proteins can regulate gene transcription by recruiting chromatin-modifying complexes to gene promoters through direct protein-protein interactions. For example, we have previously documented that in response to leptin signaling in the mouse hypothalamic AgRP neurons, TET3, via direct interaction with the transcription factor STAT3, targets a transcriptional corepressor complex containing NCOR1 and HDAC4 to the promoter of *Agrp* to inhibit transcription (80). We have also reported that in skeletal muscle cells, TET3 forms a protein complex with the peroxisome proliferator–activated receptor-γ coactivator α (PGC-1α) and interferes with its phosphorylation, leading to its destabilization (39). Dysregulation of expression/activity of PGC-1α has been associated with insulin resistance and type 2 diabetes (39). In the current work, we provide critical mechanistic insights into TET3-mediated regulation of IL-1β and IL-6, 2 key cytokines with well-established roles in endometriosis. Given the extensive global gene expression changes in response to TET3 siRNA knockdown in TET3 OE macrophages (Supplemental Figure 10, A–C), we are certain that other TET3 mechanisms must be involved. Future studies including genome-wide DNA methylation/5-hydroxymethylation profiling and elucidation of the mode of action of TET3 (e.g., enzymatic dependent versus enzymatic independent) will be necessary and important to achieve a comprehensive understanding of TET3’s role in regulation of macrophage function and related inflammatory diseases.

As a small molecule (297 Da), Bc induces TET3 degradation in a VHL-dependent fashion (Figure 4, D and E). Bc promotes the assembly of a protein complex containing VHL and TET3 (Figure 4F), suggesting that Bc might operate through a mechanism resembling that of a “molecular glue.” Molecular glues are small molecules (<500 Da) that bind to and reshape the interface surface of an E3 ligase receptor (e.g., VHL) or its target protein (e.g., TET3), thereby significantly enhancing the affinity of the 2 proteins for each other, leading to degradation of the target protein (81–84). While more extensive studies are clearly needed to elucidate the exact mechanism of action of Bc, we observe that Bc-induced TET3 degradation in a VHL-dependent manner is particularly intriguing and important in light of a recent study on small-molecule anticancer agents. ABT263 is a small-molecule inhibitor of BCL-X_L_, a well-established cancer target. However, the utility of ABT263 as an anticancer drug was limited because it induced a dose-dependent rapid thrombocytopenia, an on-target toxicity due to inhibition of BCL-X_L_ in platelets. This on-target dose-limiting toxicity was reduced after ABT263 was converted into a PROTAC that targets BCL-X_L_ for degradation through recruiting the VHL E3 ligase (85). Notably, BCL-X_L_ and VHL were both highly expressed in tumor cells, but not in platelets (85). Employing a strategy that involves utilizing a cell-specific E3 ligase to reduce on-target drug toxicities is particularly critical when aiming to target macrophages that overexpress TET3. In the Ctrl group, approximately 20% of macrophages exhibited overexpression of VHL, yet the majority of these did not show overexpression of TET3 (Supplemental Figure 18). In contrast, approximately 80% of TET3 OE macrophages also overexpressed VHL in the Endo group (Supplemental Figure 18). Notably, treating human MDMs and mouse PMs with TGF-β1 increased expression of both TET3 and VHL (Supplemental Figure 19). Thus, the striking disease-specific cooverexpression of TET3 and VHL would likely increase the specificity of Bc action and explain, at least in part, the high efficacy and low toxicity of Bc in our mouse endometriosis therapy studies (Figure 7 and Supplemental Figures 16 and 17).

Our IHC approach, which employs dual staining for CD163/TET3, does not enable the identification of all pathogenic macrophages that are likely heterogeneous in terms of developmental origins, molecular features, and surface/intracellular markers. However, this concern is mitigated by our complementary approaches highlighted below. First, an analysis of the human scRNA-Seq from an independent study (6) demonstrated a significant rise in the proportion of TET3 OE macrophages in endometriosis compared with normal controls. Specifically, using either single CD163 or a combination of markers, an 8-fold increase in the percentage of TET3 OE macrophages was observed (Figure 1, B and C, and Supplemental Figure 1). Second, our findings indicate that TET3 OE macrophages are induced by the disease microenvironment (Figure 1, Figure 2, and Supplemental Figures 4 and 8) and that TET3 overexpression is required for the viability of these cells (Figure 3 and Figure 4, G–J). While it is yet to be determined which origins of macrophages (eutopic endometrial tissue, infiltrating LPMs, and/or monocytes) undergo TET3 overexpression during endometriosis in mice, the myeloid-specific *Tet3*-KO is anticipated to eliminate the majority of TET3 OE macrophages irrespective of their developmental origins, other molecular features, and surface/intracellular markers, with the exception of those from donor mouse endometrium. Consistently, when using CD163 as a readout for macrophages, we observed a significant depletion of CD163^+^ TET3 OE macrophages in *Tet3*-KO mice while CD163^+^ TET3-negative macrophages remained unaffected (Figure 6C). We also provide in vivo evidence that these TET3 OE macrophages are proinflammatory (Figure 6, D and E). Furthermore, the KO mice exhibited decreased endometriosis burden (Figure 6, A and B). It is important to mention one limitation of our study that involves using the *LysM-cre* strain to manipulate endogenous macrophages, as LysM is also expressed in other myeloid lineage cells including granulocytes and dendritic cells (DCs) (66). Third, Bc recapitulated the therapeutic effects of myeloid-specific *Tet3*-KO (Figure 7), which was not surprising given its ability to induce apoptosis of TET3 OE macrophages (Figure 4, G–J). One would expect that Bc targets and eliminates TET3 OE macrophages (including those originating from donor mouse endometrium) irrespective of their developmental origins, other molecular features, and surface/intracellular markers. Taking into account the aforementioned factors, we conclude that the TET3 OE macrophages are pathogenic, even though they may exhibit heterogeneity in other features. Importantly, by employing the same marker (CD163) to identify macrophages in human and mouse tissues, our findings provide substantial value for translational research. Moreover, the remarkably similar therapeutic outcomes observed with myeloid-specific *Tet3* deletion and Bc treatment (Figure 6 and Figure 7) strongly bolster our conclusion (Figure 9) and mitigate concerns of potential off-target effects of Bc.

During homeostasis, the mouse peritoneal cavity contains approximately 90% LPMs of embryonic origin and approximately 10% monocyte-derived SPMs (17). Disease-associated factors induce TET3/CD163 double-positive cells in approximately 80% of human MDMs (Figure 2, B, D, and F) and in approximately 90% of mouse PMs (Supplemental Figure 8, B, D, and F). This indicates that both embryonic and MDMs can be induced to overexpress TET3 under disease conditions. In human endometriosis lesions, TET3 OE macrophages were predominantly found in 2 subpopulations that expressed some markers of “tissue-resident macrophages/PMs,” but did not express GATA6, a signature marker of LPMs of embryonic origin (Supplemental Figure 2) (6, 17). Several possibilities can be envisioned. First, the TET3 OE macrophages in human endometriosis lesions were of embryonic origin but lost GATA6 expression due to reprogramming by the disease microenvironment. This is consistent with the findings from mice where embryonic-derived LPMs stopped expressing GATA6 after infiltrating endometriosis lesions (10). Second, the TET3 OE macrophages in human endometriosis lesions were of monocyte origin, but were reprogrammed by the disease microenvironment to express some embryonic-lineage markers. Third, the TET3 OE macrophages in human endometriosis lesions were a mixture of both embryonic and monocyte origins. Nonetheless, the key finding from our work is that the TET3 OE macrophages are a distinct population that are pathogenic, regardless of their developmental origins, and that they can be specifically targeted to eliminate the disease. Finally, as TET3 OE macrophages are induced by the inflammatory microenvironment, we propose the possibility of employing TET3 degraders as potential therapeutic agents for other chronic inflammatory diseases.

## Methods

Additional methods details can be found in Supplemental Methods.

### Sex as a biological variable.

As endometriosis is relevant only to females, only female mice and humans were involved in the present studies.

### Human samples.

Human PBMCs were collected and enriched by density gradient centrifugation. Macrophage differentiation was induced by treating PBMCs with recombinant human M-CSF (Gibco, Thermo Fisher, PHC9504). Briefly, 15 mL of blood samples were taken from voluntary donors in sterile EDTA (K2) tubes (BD, catalog 366643) and diluted 1:1 in PBS. Diluted samples (15 mL) were laid onto 15 mL of Ficoll-Paque PLUS density gradient medium (Cytiva, 17144003) and centrifuged without excel and brake at 400*g* at room temperature (RT) for 20 minutes. PBMCs were harvested from the mononuclear layer and washed twice with PBS by centrifugation at 300*g* for 8 minutes at 4°C each time. To induce differentiation to macrophages (MDMs), PBMCs were resuspended in growth media (RPMI 1640 supplemented with 10% heat-inactivated FBS) (Gibco, Thermo Fisher Scientific, 16140-017), 1% antibiotic-antimycotic (Gibco, Thermo Fisher Scientific, 15240-062), and 50 ng/mL of M-CSF, seeded in 12-well plates at the density of 1 × 10^6^/well or 24-well plates at the density of 2.5 × 10^5^/well and maintained at 37°C in a 5% humidified CO_2_ tissue culture incubator. Seven days later, nonadherent cells were removed and media were replaced with new growth media every 3 days. To induce macrophage polarization, M-CSF–induced MDMs were treated with IL-4 (204-IL-010, R&D) at 20 ng/mL for 48 hours, followed by further experiments. Formalin-fixed, paraffin-embedded (FFPE) human tissue blocks of normal endometrium (*n* = 5) and i.p. endometriosis lesions (*n* = 7) were obtained from the Yale Pathology Tissue Services and Nanjing Drum Tower Hospital, The Affiliated Hospital of Nanjing University Medical School. Patient characteristics are shown in Supplemental Table 1.

### Bc treatment of cultured human and mouse macrophages.

Bc powder (Sigma-Aldrich, SML2611) was freshly dissolved in DMSO at a concentration of 5 mM and filtered through a 0.22 micron. For cultured MDMs, cells were primed with CM-Endo or TGF-β1 (PeproTech, 100-21) for 36–48 hours (to increase TET3 expression) before Bc treatment. For cultured mouse PMs, cells were primed with 30 ng/mL of TGF-β1 (R&D Systems, 7666-MB0-005) for 48 hours (to increase TET3 expression) before Bc treatment. Bc (or DMSO) was added at a final concentration of 10 μM, followed by RNA and protein extraction at time points indicated in the figure legends. For TET protein stability assay of MDMs, cells in 24-well plates were incubated with Veh or Bc at a final concentration of 10 μM for 3 hours, followed by addition of CHX (Cell Signaling, 2112) at a final concentration of 50 μg/mL. Proteins were harvested at 0, 2, 4, and 6 hours after addition of CHX. For TET3 expression restoration experiments, cells seeded in 24-well plates were infected with Ad-GFP or Ad-TET3 at 4000 gc/cell. Following 16 hours of infection, Veh or Bc was added at a final concentration of 10 μM.

### VHL and Flag-TET3 co-IP and Western blot analysis.

Anti-Flag magnetic agarose beads (Invitrogen, Thermo Scientific, A36797) 30 μL were washed twice with 1 mL of IP buffer (0.5% Triton X-100, 150 mM NaCl, 10 mM Tris-HCl at pH 7.5, and 10 mM EDTA) and kept on ice until use, with 1 tube saved as IgG. To prepare lysate from H1299 (ATCC, CRL-5803) cells, cells at a density of 5 × 10^6^ cells/well in a 100 mm plate were infected with Ad-TET3 adenovirus (Ad-FLAG.h-TET3, ADV-225322, Vector Biolabs) at 5 × 10^5^ PFU/mL for 48 hours; then the cells were treated with Veh or Bc (50 μM) for 2 hours. Cells were then rinsed with cold PBS 3 times, collected by manual scraping in cold PBS, and pelleted by gentle centrifugation at 300*g* at 4°C for 5 minutes. The cell pellet was resuspended in 1 mL of cold freshly prepared gentle lysis buffer (GLB, 0.5% Triton X-100, 10 mM NaCl, 10 mM Tris-HCl at pH 7.5, 10 mM EDTA, and 1× protease inhibitor cocktail) and incubated on ice for 20 minutes with occasional inversion. After centrifugation at 12,000*g* at 4°C for 15 minutes to remove insoluble materials, 5 M of NaCl was added to a final concentration of 200 mM, and the lysate was transferred to a tube containing anti-DYKDDDDK magnetic agarose (850 μL of lysate per IP). IP was carried out at 4°C for 4 hours. Following IP, beads were quickly washed twice with 1 mL of cold IP buffer and washed an additional 3 times by rotating at 4°C for 5 minutes each time. After the final wash, residual liquid was completely removed and the beads were eluted with 30 μL of 2xSDS buffer (containing 1× phosphatase inhibitor cocktail and 1× protease inhibitor cocktail) at 100°C for 5 minutes. Eluant was loaded (10 μl per gel well) onto a 4%–15% gradient SDS gel (Bio-Rad, 456-8086). For Western blot analysis, anti-TET3 (Cell Signaling Technology, 99980) and anti-VHL (Proteintech, 24756-1-AP) were used. The secondary antibody used was HRP-linked anti-rabbit IgG (dilution 1:10,000; Rockland, 611-1322).

### Proinflammatory activation of macrophages and measurements of IL-1β and IL-6 proteins.

Proinflammatory activation of PMs and MDMs was achieved by treatment with a combination of 10 ng/mL of LPS (Invitrogen, 00-4976-93) and 20 ng/mL of IFN-γ (R&D Systems, 485-MI-100). RNAs were isolated at time points indicated in figure legends. IL-1β and IL-6 protein levels in the supernatant of cultured mouse PMs were measured using ELISA kits (R&D Systems, MLB00C and M6000B-1). For IL-1β, LPS/IFN-γ–primed mouse PMs were incubated with 5 mM ATP (Alfa Aesar, L14522) for 30 minutes before collection of supernatants for ELISA analysis. Supernatants of cultured mouse peripheral macrophages were collected by centrifugation at 1,000*g* at 4°C for 20 minutes to remove cell debris. To measure IL-1β protein levels using an ELISA kit (Elabscience, E-EL-H0149c), cell lysates of human MDMs were collected. To measure IL-6 protein levels using an ELISA kit (E-EL-H6156), supernatants of human MDMs were collected. To obtain cell lysates, MDMs of 1 × 10^6^ in each sample were digested with Trypsin-EDTA (0.25%), followed by centrifugation at 1,000*g* for 5 minutes. The supernatants were discarded, and cell pellets were suspended with 150 μL PBS containing protease inhibitors. The resulting cell suspension was incubated in liquid nitrogen for 30 minutes followed by rapid thawing in a 37°C water bath. The above steps were repeated 3 times. The cell lysate was cleared by centrifugation at 1,500*g* for 10 minutes at 4°C to remove insoluble materials. IL-1β and IL-6 protein concentrations were presented after normalization against numbers of viable cells.

### Induction of endometriosis.

Endometriosis in mice was surgically induced using our previously described methods (86–88). Briefly, uterus horns were removed from WT female donor mice (7 weeks old), opened longitudinally, and cut into fragments of 3 mm. Two uterine segments were sutured to each right and left parietal peritoneum of recipient mice (7–8 weeks old) with absorbable suture. Sham surgeries were performed for the sham group using the same surgical procedure without the introduction of extraneous uterine tissue. The experimental model was allowed to develop for more than 4 weeks. Development of the model was confirmed by opening the abdominal cavity and measuring the size of endometriotic lesions both macroscopically and histologically.

### Statistics.

The statistical analyses for each figure are indicated in the legends. All statistical analyses were performed using GraphPad Prism, version 8, for Windows (GraphPad Software) and are presented as mean ± SEM. Two-tailed Student’s *t* tests (or as otherwise indicated) were used to compare means between groups. *P* < 0.05 was considered significant.

### Study approval.

The present studies in mice and studies involving using human blood samples were reviewed and approved by the Yale University Institutional Animal Care and Use Committee and Yale University Human Investigation Committee.

### Data availability.

All study data are included in the article, supplemental information, and the Supporting Data Values file. Bulk RNA-Seq data are available at the NCBI’s Gene Expression Omnibus (GEO GSE223106).

## Author contributions

Y Huang conceived the project and designed and wrote the manuscript. HL, BL, YD, FL, and YZ carried out experiments. HL and BL analyzed data and helped with manuscript preparation. SB performed flow cytometry analysis. DZ performed bulk RNA-Seq analysis. RM contributed technical expertise in endometriosis mouse models. MV provided intellectual insights into small molecule–induced protein degradation. Y Hu, DL and HST provided important intellectual insights into endometriosis. GGC provided critical discussions throughout the work.

## Supplementary Material

Supplemental data

Unedited blot and gel images

Supporting data values

## Version 1 08/14/2024

In-Press Preview

## Version 2 11/01/2024

Electronic publication
