# Supplementary material for: TET3-overexpressing macrophages promote endometriosis
Source: J Clin Invest. 2024 Nov 1;134(21):e181839. doi: 10.1172/JCI181839 (PMC11527447; doi:10.1172/JCI181839)
Supplement: Supplemental data [file jci-134-181839-s078.pdf]

Supplemental Information for

TET3-overexpressing macrophages promote endometriosis

Haining Lv[1,2 #], Beibei Liu[1,3#], Yangyang Dai[1,4], Feng Li[1,5], Stefania Bellone[1], Yuping Zhou[1], Ramanaiah Mamillapalli[1], Dejian Zhao[6], Muthukumaran Venkatachalapathy[7], Yali Hu[2], Gordon G. Carmichael[8], Da Li[1,3*], Hugh S. Taylor[1*], Yingqun Huang [1,9*]

Files:

Supplemental Methods

Supplemental Figures 1-19

Supplemental Tables 1-3

Supplemental Methods

Mouse

All animal work was approved by the Yale University Institutional Animal Care and Use Committee. Mice were housed at 22°C-24°C with a 12 h light/12 h dark cycle with a standard chow diet (Harlan Teklad no. 2018, 18% calories from fat) and water provided ad libitum. Female C57BL/6J (JAX, 000664) mice were purchased from the Jackson Laboratory. *Tet3*$^{fl/fl}$ mice were kindly provided by Anjana Rao from La Jolla Institute for Immunology. Mye-Tet3 ko mice (*Lysm*$^{+/wt}$*Tet3*$^{fl/fl}$) were generated by crossing LysM-cre (JAX, 004781, Jackson Laboratory) and *Tet3*$^{fl/fl}$ mice. Littermate *Lysm*$^{wt/wt}$*Tet3*$^{fl/fl}$ mice were used as control (WT). For all experiments, age-matched animals were used. For information on animal numbers, refer to figure legends.

Bobcat339 treatment of mice

Bobcat339 powder was freshly dissolved in DMSO at a concentration of 50 mg/mL and filtered through a 0.22-micron filter. The stock was freshly diluted with 1xPBS to 0.5 mg/ml before injection into mice.

Analysis of human scRNA-seq data

We extracted and analyzed the data of GSM6102532, GSM6102533, GSM6102534, GSM6595248, GSM6595250, GSM6595252, GSM6595261, GSM6102562, and GSM6102556 (Supplemental Table 2) (*1*). Raw data were processed according to 10x genomics workflow. The output files were imported into the Seurat (4.0.5) R toolkit to construct Seurat objects. The raw gene expression measurements for each cell were normalized by dividing them by the total expression followed by scale factor-multiplying (x 10,000) and log-transformation (UMI-per-10,000+1) in the Seurat toolkit. Cells that expressed fewer than 200 genes or mitochondrial gene content > 15% of the total UMI count were excluded. The normalized gene expression matrix generated after data preprocessing was used to identify the major cell clusters by applying dimension reduction and clustering. Uniform Manifold Approximation and Projection (UMAP) method was used for visualization of unsupervised clustering at the resolution of 0.5. Identification of the cellular subsets and their marker genes referred to published article (*1*). After identifying all cell types, macrophages of the dataset were captured to show TET3, CD163 and VHL expression and distribution between control and disease groups in UMAP. The cut-off for TET3 and CD163 OE was set at 1.5. Top feature genes for tissue-resident macrophages, peritoneal macrophages and other macrophage sub-populations were selected based on (*1*). The expression values of these feature genes in TET3 OE macrophages were presented by VlnPlot function in Seurat Toolkit. Patient information is presented in Supplemental Table 2 (*1*).

Cell lines and treatments

Mouse macrophage cell line Raw 264.7 (ATCC, TIB-71) was purchased. The human endometriosis stromal cell line and normal human endometrial stromal cell line (HESC) were previously described (*2*). Raw 264.7 cells were maintained in DMEM (Gibco, 11965-092) supplemented with 10% FBS (ATCC, 30-2020) and 1% Anti-Anti. Human normal and endometriosis cell lines were maintained in DMEM-F12 (Gibco, 11330-032) containing 10% FBS and 1% Anti-Anti.

Conditioned media preparation and treatment

To prepare CM-Endo and CM-HESC, immortalized human endometriosis stromal cells or human normal endometrial stromal cells were seeded in 24-well plates (2.5 x 10^5 cells/well) with growth media and allowed to grow to ~50% confluency. Cells were then washed with PBS and incubated with fresh growth media (500 µl/well) for an additional 24 h. Cell supernatant (CM-Endo and CM-HESC) was collected and immediately used to treat MDMs after 1:1 dilution with growth media. In the control group, only growth media were used.

Cell transfection

For siRNA transfection in a 24-well plate scale of Raw 264.7 cells, 10 pmol of NT siRNA (AM4636, Ambion) or *Tet3* siRNA (4390815/s101483, Ambion) was mixed with 100 µL of OPTI-MEM (Gibco, 31985-070) by gentle pipetting. In parallel, 3 µL of Lipofectamine RNAiMAX (Invitrogen, 13778-150) was mixed with 100 µL of OPTI-

MEM by gentle pipetting. Following 5 min of incubation at RT, the two were combined and the resulting 200 µL of transfection solution was added to each well of cells. After 24 h of incubation at 37^0C in a 5% humidified CO2 tissue culture incubator, 300 µL of growth media was added and incubation was continued until cell harvesting. For siRNA transfection in a 24-well plate scale of MDMs, NT siRNA or TET3 siRNA (4392420/s47239, Ambion) was mixed with 25 µL of OPTI-MEM by gentle pipetting. In parallel, 1.5 µL of Lipofectamine RNAiMAX was mixed with 25 µL of OPTI-MEM by gentle pipetting. Following 5 min of incubation at RT, the resulting 50 µl of transfection solution was added to each well of cells containing 450 µL of growth media. For let-7a transfection of mouse peritoneal macrophages (PM) in a 24-well plate scale, 10 pmol let-7a (let-7a-5p mimic, Active Motif, MIM0001) or miCon (non-targeting miRNA mimic, Active Motif, MIM9001) was mixed with 100 µL of OPTI-MEM by gentle pipetting. In parallel, 3 µL of Lipofectamine RNAiMAX was mixed with 100 µL of OPTI-MEM by gentle pipetting. Following 5 min of incubation at RT, the resulting 200 µl of transfection solution was added to each well of cells. Following incubation in a CO2 tissue culture incubator for 6 h, 200 µL of growth media were added and incubation was continued to the next day until further treatments with LPS/IFN-γ.

For TET3 overexpression in MDMs, MDMs seeded in 24-well plates at 2×10^5 cells / well were infected with Ad-GFP (Control adenovirus, 1060, Vector Biolabs) or Ad-TET3 (Ad-FLAG.h-TET3, ADV-225322, Vector Biolabs) at $5x10^6$ PFU/ml for 24 h. These were followed by RNA extraction or further treatments with LPS/IFN-γ.

qRT-PCR

Total RNAs were extracted from cells using PureLink RNA Mini Kit (Invitrogen, 12183025). 0.5-1 µg total RNA was reverse transcribed to cDNA in a reaction volume of 20 µL using PrimeScript RT Reagent Kit (TaKaRa, RR037B). Real-time quantitative PCR was performed in a 15 µL reaction volume containing 0.5–1 µL of cDNA using SsoAdvanced Universal SYBR Green Supermix in a Bio-Rad iCycler. Gene expression levels were normalized against RPLP0. The specific PCR primers were summarized in Supplemental Table 3.

For let-7a qPCR, 10 ng of total RNA was reverse transcribed to cDNA in a reaction volume of 10 µl using miRCURY LNA RT Kit (QIAGEN, 339340). Quantitative real-time PCR reactions were carried out using miRCURY LNA SYBR Green PCR Kit (QIAGEN, 339346) and a let-7a-specific primer (Has-Let-7a-5p miRCURY LNA miRNA PCR Assay, QIAGEN, 339306/YP00205727) in a Bio-Rad iCycler. Gene expression levels were normalized against U6 (U6 snRNA v2 miRURY LNA miRNA PCR Assay, QIAGEN, 339306/YP02119464, QIAGEN).

Western blot analysis

Cells were homogenized in situ using a pipette tip in 2x SDS-sample buffer with 10% β-mercaptoethanol at RT followed by heating at 100 °C for 5 min with occasional vortexing. The samples were loaded onto a 4-15% gradient SDS gel (Bio-rad, 456-8086) (5-10 µL/well) and transferred to nitrocellulose membranes, followed by Western blot analysis. The antibodies used were anti-TET3 (diluted at 1:1000, GeneTex, GTX121453 for mouse peritoneal macrophages; diluted at 1:000, Cell Signaling Technology, 99980 for human MDMs, RAW 264.7 and H1299 cells), anti-VHL (diluted at 1:1000, Proteintech, 24756-1-AP for human MDMs, diluted at 1:1000, Invitrogen, Thermo Scientific, PA5-27322 for mouse peritoneal macrophages), anti-TET2 (diluted at 1:1000, Proteintech, 21207-1-AP for human/mouse), and HRP-conjugated anti-GAPDH (dilution 1:5000; Proteintech, HRP-60004). The secondary antibody was HRP-linked Anti-rabbit IgG (dilution 1:10,000; Rockland, 611-1322).

TUNEL Assays

These were performed using the In Situ Cell Death Detection Kit (Sigma Aldrich, 12156792910). Briefly, cells were fixed with 4% paraformaldehyde in PBS for 1 h at RT and rinsed with PBS, followed by permeabilization with 0.1% Triton X-100 in 0.1% sodium citrate for 2 min on ice. TUNEL reaction mixture was added and

incubation was carried out at 37 °C for 60 min. DAPI was added to counter-stain the cells for 1 min. The slides were coverslipped and scoped using a Keyence BZ-X700 fluorescence microscope.

Flow cytometry
Total bone marrow cells were harvested from femur bones of female mice into the FACS buffer (0.5% FBS in PBS). Cells from spleen were harvested after gently macerating the spleens by a sterile 3 mL syringe plunger in the FACS buffer. Cell suspension was filtered through a 70 µm nylon strainer, centrifuged and resuspended in 5 mL of red blood cell lysis buffer (BioLegend, 420301) to exclude any red blood cells. The cells were resuspended in FACS buffer and stained by antibodies for 15 min in the dark at RT. The antibodies used were CD11b-PE (diluted at 1:100, BioLegend, 101208), NK1.1-APC (diluted at 1:50, BioLegend, 108710), Ly6C-PerCP (diluted at 1:100, BioLegend, 128028) and Ly6G-FITC (diluted at 1:200, BioLegend, 127606). Data were acquired using BD FACSCalibur flow cytometry system and analysis was performed using Flowjo V10.

Mouse peritoneal macrophage collection
Peritoneal macrophages were collected from the peritoneal cavity of WT or Mye-Tet3 ko mice by injecting 10 mL of PBS followed by gentle abdominal massaging for 5 min. Cells were pelleted by centrifugation at 1500 x g at 4 °C for 8 min. To remove red blood cells, cells were resuspended in RBC Lysis Buffer (Biolegend, 420302, diluted to 1X) and allowed to stand for 5 min, followed by addition of PBS to stop the reaction and twice wash with PBS by centrifugation. Macrophages were enriched by plating cells in RPMI 1640 supplemented with 10% FBS and 1% Anti-Anti for 2 h at 37°C in a 5% humidified CO2 tissue culture incubator. Non-adherent cells were then removed with three PBS washes.

Blood chemistry
Blood samples were collected in EDTA tubes (Microtainer with K2EDTA, BD, 365974) by cardiac puncture of terminally anesthetized animals. The tubes were centrifuged at 2,000 x g at 4 °C for 20 min, and plasma was collected and stored at -80 °C until use. Blood chemistry were measured according to the manufacturer's instructions. Kits used to measure alanine transaminase (EALT-100) and aspartate transaminase (EASTR-100) were purchased from Bioassay Systems. The bilirubin assay kit (MAK126) was purchased from Sigma Aldrich.

VHLprotac treatment of human MDM and mouse PM
VHLprotac powder (MedChemExpress, HY-111593) was dissolved in DMSO to make a stock solution of 5 mM and filtered through a 0.22 micron. To make working solution, the stock was freshly diluted with respective cell culture media and used at a final concentration of 5 μM.

RNA sequencing and data analysis
RNA-Seq library preparation and sequencing for RAW 264.7 samples were conducted at Yale Stem Cell Center Genomics Core facility through poly A enrichment (Illumina TruSeq Stranded mRNA Library Prep Kit). Differential expression analysis between two different groups was performed with DESeq2 software (https://bioconductor.org/packages/release/bioc/html/DESeq2.html). Genes with a false discovery rate (FDR) below 0.05 and absolute fold change over 1.0 were analyzed with Ingenuity Pathway Analysis using IPA software (Qiagen).

Fluorescent immunohistochemistry (IHC) of tissue sections
FFPE tissue slides were deparaffinized by heating at 65°C for 60 min, followed by xylene wash 3 times 5 min each. The slides were then rehydrated in 100%, 90%, 80% and 70% ethanol for 5 min each, followed by ddH_2O wash 2 times 5 min each. For antigen retrieval, slides were heated to near boiling in sodium citrate solution (freshly prepared, pH 6.0) for 2 min, followed by incubation in a steamer (BELLA Food Steamer, Model # XJ-10102A) for 15 min. The slides were cooled at RT to below 40°C, followed by sequential wash in PBS and PBS-T (0.05% Tween 20/PBS) for 5 min each. After permeabilization in 1% Triton X-100/PBS for 15 min,

slides were blocked in 5% donkey serum/PBS-T at RT for 1 h and quickly washed once with PBS-T before antibody incubation. For double-staining of CD163/TET3, CD163/IL-1β, and CD163/IL-6, slides were incubated with antibodies diluted in 2% donkey serum/PBS-T overnight at 4°C. Antibodies used are anti-TET3 (GeneTex, GTX121453, diluted at 1:400 for human tissue; Millipore Sigma, ABE290, diluted at 1:500 for mouse tissue), anti-CD163 (Abcam, ab156769, diluted at 1:400 for human tissue; Santa Cruz Biotechnology, sc-58965, diluted at 1:200 for mouse tissue), anti-IL-1β (Proteintech, 26048-1-AP, diluted at 1:200), and anti-IL-6 (Abcam, AB6672, diluted at 1:200). Negative controls were performed by omitting the respective primary antibodies. The next day, slides were washed 3 times and incubated in 0.4% Triton X-100/PBS with the secondary antibody donkey anti-Rabbit IgG Fluor 594 (Invitrogen, A-21207, diluted at 1:500), goat anti-Mouse IgG Fluor 488 (Invitrogen, A-11029, diluted at 1:500), and goat anti-Rat IgG Flour 488 (Abcam, ab150157, diluted at 1:500) for 1 h at RT. The slides were covered with antifade mounting medium with DAPI (Vector laboratories, H-2000), scoped using a Keyence BZ-X700 fluorescence microscope.

Fluorescent immunocytochemistry (ICC) of human MDMs and mouse peritoneal macrophages

After wash in cold PBS, adherent cells were fixed with 4% paraformaldehyde in PBS for 15 min and permeabilized with 0.4% TritonX-100 for 15 min. Cells were blocked in 5% donkey serum/PBS-T at RT for 30 min and quickly washed once with PBS-T before antibody incubation. Primary antibodies were diluted in 2% donkey serum/PBS-T. For CD163/TET3 double staining, cells were incubated with anti-TET3 (GeneTex, GTX121453, diluted at 1:400 for human MDMs; Millipore Sigma, ABE290, diluted at 1:500 for mouse peritoneal macrophages) and anti-CD163 (Abcam, ab156769, diluted at 1:400 for MDMs; Santa Cruz Biotechnology, sc-58965, diluted at 1:200 for peritoneal macrophages) overnight at 4°C. The next day, cells were washed 3 times with PBS-T and incubated in 0.4% Triton X-100/PBS with donkey anti-Rabbit IgG Fluor 594 (Invitrogen, A-21207, diluted at 1:500) and goat anti-Mouse IgG Fluor 488 (Invitrogen, A-11029, diluted at 1:500) for 1 h at RT. Cells were covered with antifade mounting medium with DAPI (Vector laboratories, H-2000), scoped using a Keyence BZ-X700 fluorescence microscope.

Immunofluorescent staining quantification

Image analysis and fluorescent signal quantification were performed and analyzed using ImageJ. Six tissue sections per mouse were quantified, with 5 mice in each group. Fluorescent signal was quantified as mean fluorescence intensity (MFI) and normalized to $CD163^{+}$ macrophage area. For cultured macrophages, 3 randomly picked fields per group were used for quantification.

Flow cytometry

Total bone marrow cells were harvested from femur bones of female mice into the FACS buffer (0.5% FBS in PBS). Cells from spleen were harvested after gently macerating the spleens by a sterile 3 mL syringe plunger in the FACS buffer. Cell suspension was filtered through a 70 µm nylon strainer, centrifuged and resuspended in 5 mL of red blood cell lysis buffer (BioLegend, 420301) to exclude any red blood cells. The cells were resuspended in FACS buffer and stained by antibodies for 15 min in the dark at RT. The antibodies used were CD11b-PE (diluted at 1:100, BioLegend, 101208), NK1.1-APC (diluted at 1:50, BioLegend, 108710), Ly6C-PerCP (diluted at 1:100, BioLegend, 128028) and Ly6G-FITC (diluted at 1:200, BioLegend, 127606). Data were acquired using BD FACSCalibur flow cytometry system and analysis was performed using Flowjo V10.

References

1. Y. Tan *et al.*, Single-cell analysis of endometriosis reveals a coordinated transcriptional programme driving immunotolerance and angiogenesis across eutopic and ectopic tissues. *Nat Cell Biol* **24**, 1306-1318 (2022).
2. P. Chen, R. Mamillapalli, S. Habata, H. S. Taylor, Endometriosis Cell Proliferation Induced by Bone Marrow Mesenchymal Stem Cells. *Reprod Sci* **28**, 426-434 (2021).

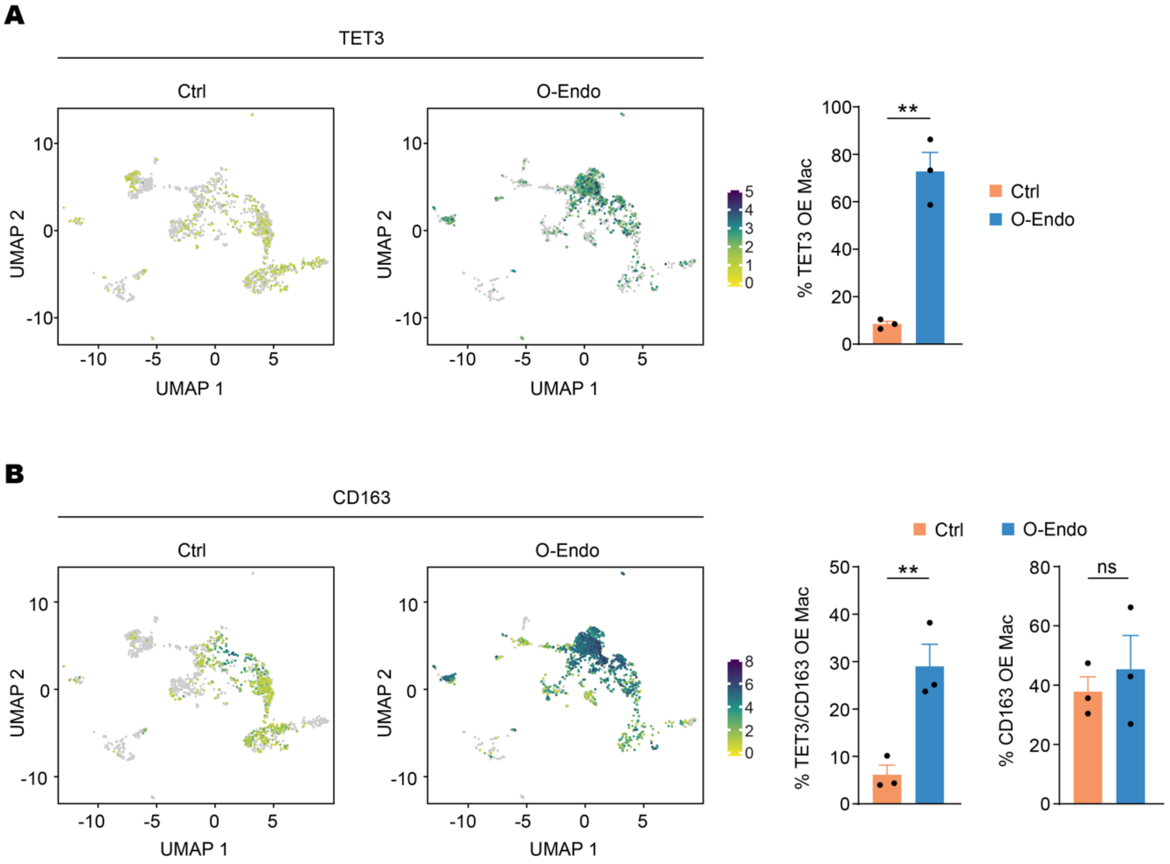

Figure S1. **TET3 OE macrophages are abundant in human ovarian endometriotic lesions.** (**A**) UMAP showing macrophage TET3 expression in Ctrl (*n* = 3) and ovarian endometriosis (O-Endo, *n* = 3), with bar graph displaying percentages of TET3 OE macrophages on the right. (**B**) UMAP showing macrophage CD163 expression in Ctrl (*n* = 3) and O-Endo (*n* = 3). Bar graphs on the right show percentages of CD163 OE and TET3/CD163 double OE macrophages. Data represent the mean ± SEM. $^{**}P < 0.01$ and $^{***}P < 0.001$, by 2-tailed Student's *t* test.

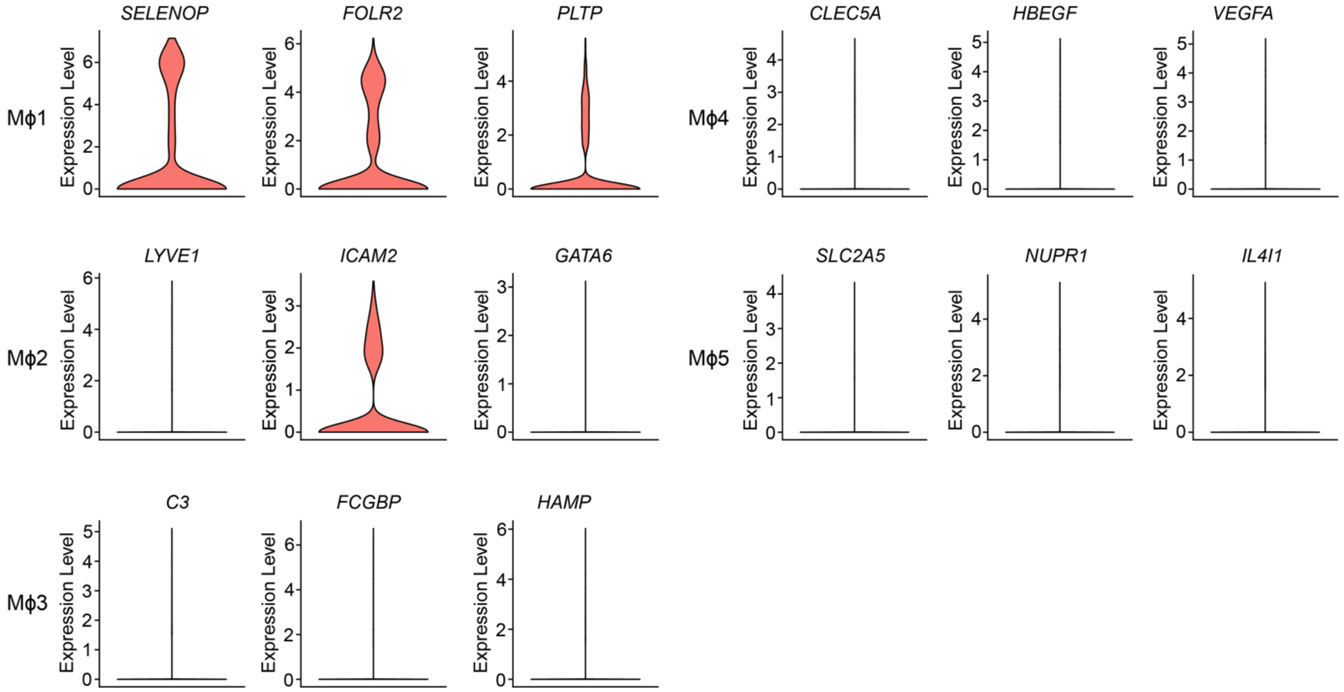

Figure S2. Violin plots showing relative expression values of indicated genes of the five macrophage subpopulations identified in human peritoneal and ovarian endometriotic lesions in TET3 OE macrophages. TET3 OE macrophages were predominantly detected in the MΦ1 and MΦ2 categories.

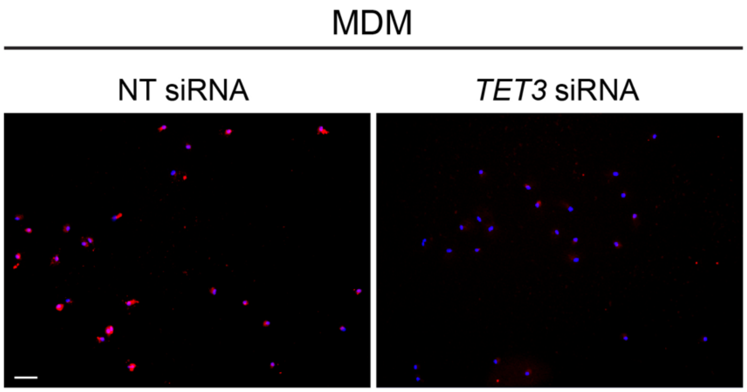

Figure S3. Human primary peripheral blood monocyte-derived macrophages (MDM) were transfected with *TET3* siRNA (siRNA specifically targeting human *TET3*) or NT siRNA (control nontargeting siRNA) for 48 h. Immunofluorescence staining using anti-TET3 (GeneTex, GTX121453, diluted at 1:400) showed a significant reduction in TET3 (red) in *TET3* siRNA-transfected as compared to NT siRNA-transfected cells. Cell nuclei are stained blue by DAPI. MDMs were treated with TGF-β1 at a final concentration of 10 ng/ml to increase TET3 expression. Scale bar: 40 μm.

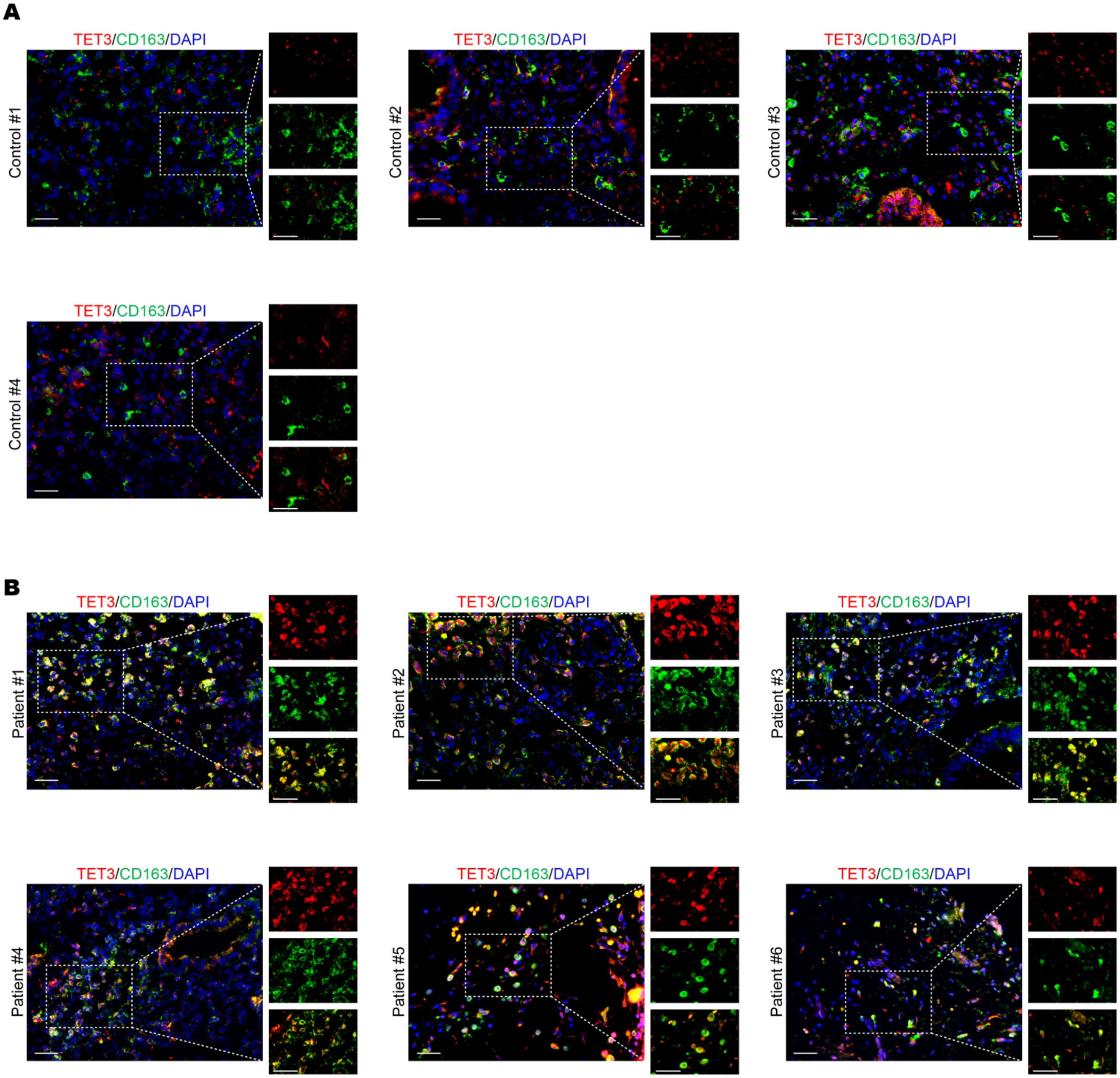

Figure S4. **TET3 OE macrophages are associated with human endometriosis.** Representative immunofluorescence staining of TET3 (red), CD163 (green) and nuclei (blue) from human normal endometrial tissue (*n* = 4) and peritoneal endometriosis tissue (*n* = 6). The panels on the right are zoomed-in images from the left. Scale bar: 40 μm.

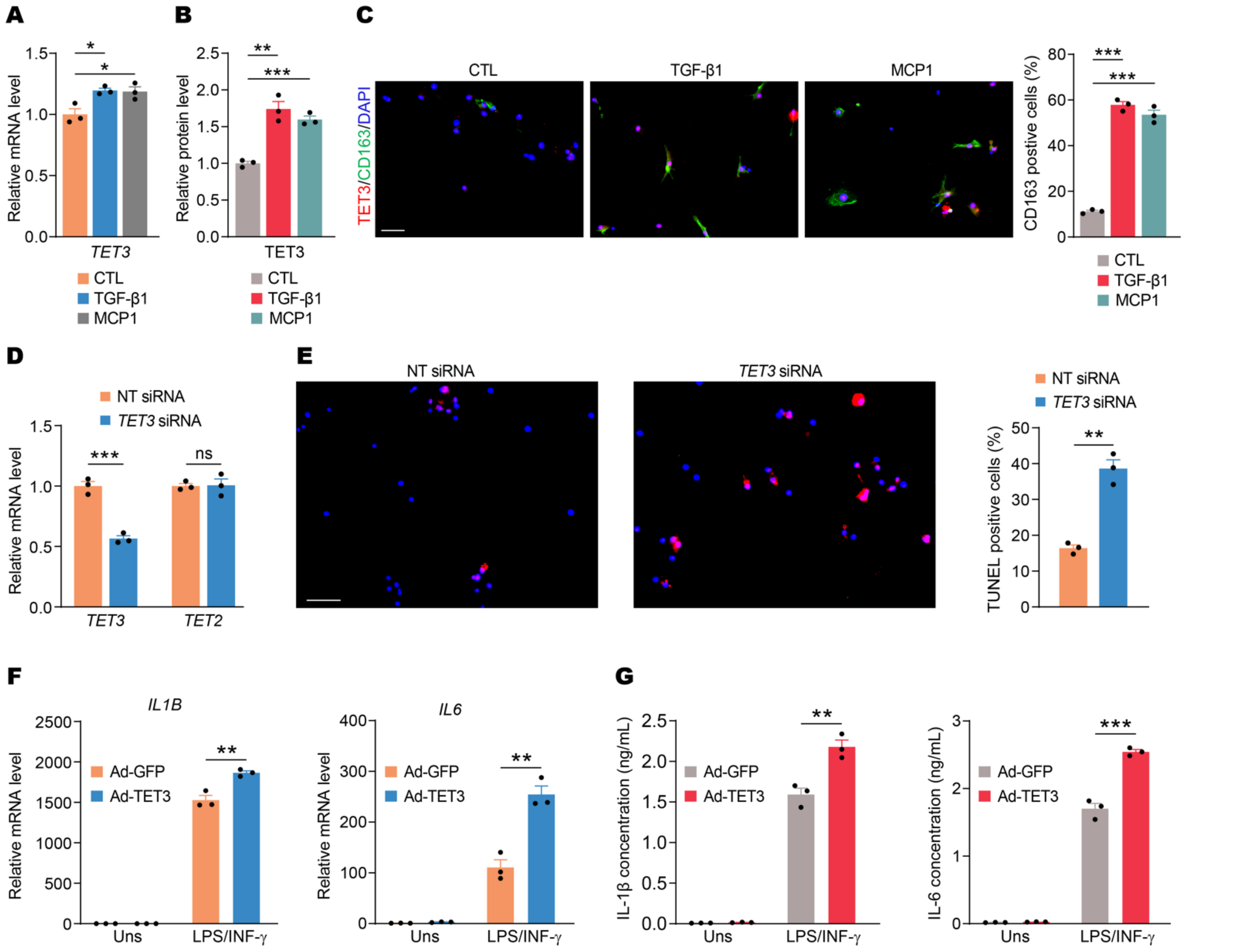

Figure S5. TET3 affects cell viability and pro-inflammatory cytokine production in human MDMs induced by M-CSF for 7 days and subsequently polarized with IL-4 at 20 ng/ml for 48 h. (**A**) MDMs were treated with CTL, TGF-β1 (10 ng/ml), or MCP1 (200 ng/ml), followed by RNA (24 h) and protein (48 h) analyses. qRT-PCR of TET3 mRNA (**A**) and IHC quantification of TET3 protein (**B**) using MFI of TET3 in **C** are shown. (**C**) Representative photomicrographs and corresponding statistical analysis of immunostaining of TET3 and CD163 in MDMs treated as in **A**. For quantification of immunostaining, *n* = 3 randomly selected areas per group were used. (**D**) qRT-PCR of TET3 and TET2 mRNAs isolated from MDMs transfected with NT siRNA or *TET3* siRNA for 24 h. *n* = 3 per group in technical replicates. (**E**) Representative photomicrographs and corresponding statistical analysis of TUNEL$^+$ (red) MDMs treated as in **D**. TUNEL assays were performed after 48 h of transfection. *n* = 3 randomly selected areas per group. (**F**) MDMs were infected with Ad-GFP or Ad-TET3. The next day, cells were stimulated with 10 ng/ml LPS plus 20 ng/ml IFN-γ. IL1β and IL6 mRNAs were isolated at 1 h and 12 h after LPS/IFN-γ stimulation, followed by qRT-PCR. *n* = 3 in biological replicates. (**G**) ELISA analysis of IL-1β (3 h) and IL-6 (12 h) of MDMs following treatment as in **F**. All data represent the mean ± SEM. **P* < 0.05, ***P* < 0.01, and ****P* < 0.001, by 2-tailed Student's *t* test. Scale bar: 40 μm.

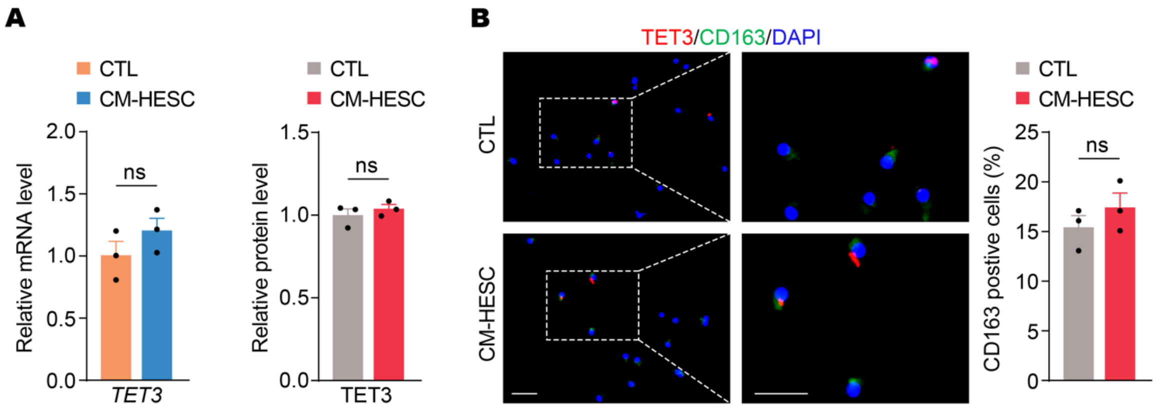

Figure S6. Conditioned media from normal endometrial stromal cells does not upregulate TET3 expression. (**A**) qRT-PCR of TET3 mRNA (left) and IHC quantification of TET3 protein (right) from MDMs treated with CTL or CM-HESC for 72 h. MFI of TET3 in **B** was used to quantify TET3 protein expression. (**B**) Representative photomicrographs and corresponding statistical analysis of immunostaining of TET3 and CD163 in MDMs treated as in **A**. For quantification of immunostaining, *n* = 3 randomly selected areas per group were used. Data represent the mean ± SEM. ns, not statistically significant, by 2-tailed Student's *t* test. Scale bar: 40 μm.

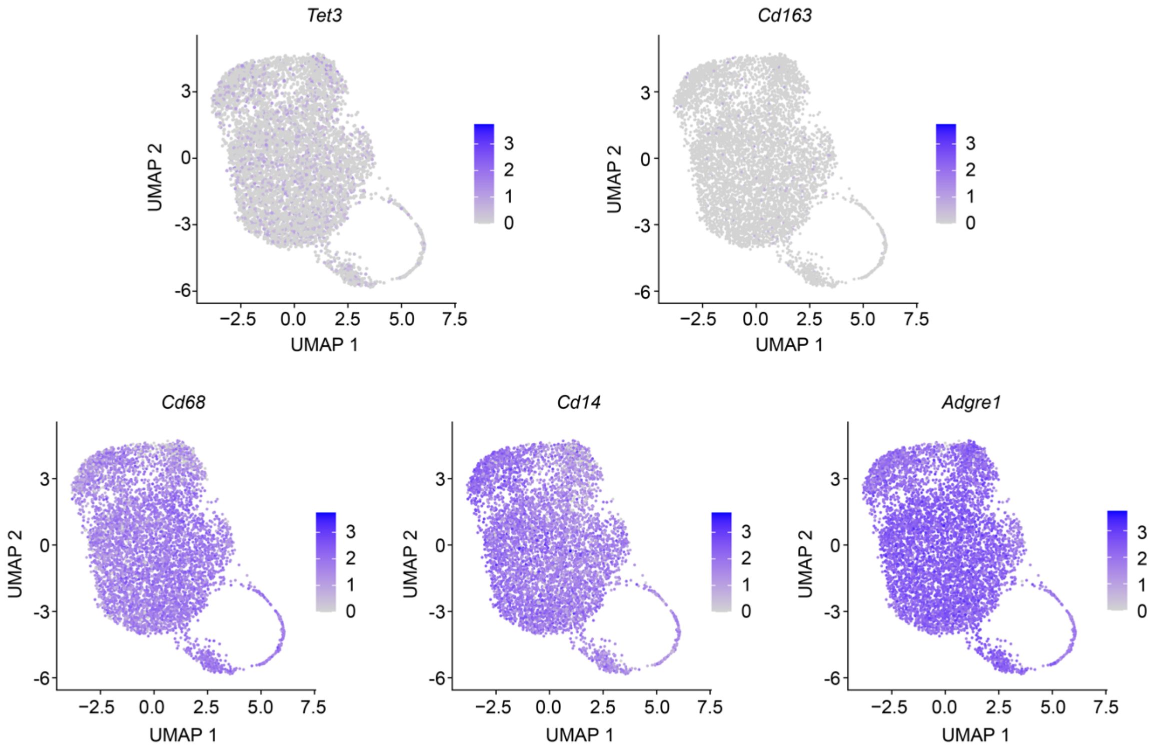

Figure S7. scRNA-Seq of peritoneal macrophages from naïve adult female mice. UMAP plots show baseline Tet3 and Cd163 expression in macrophages identified using pan-macrophage markers Cd68, Cd14 and Adgre1(F4/80).

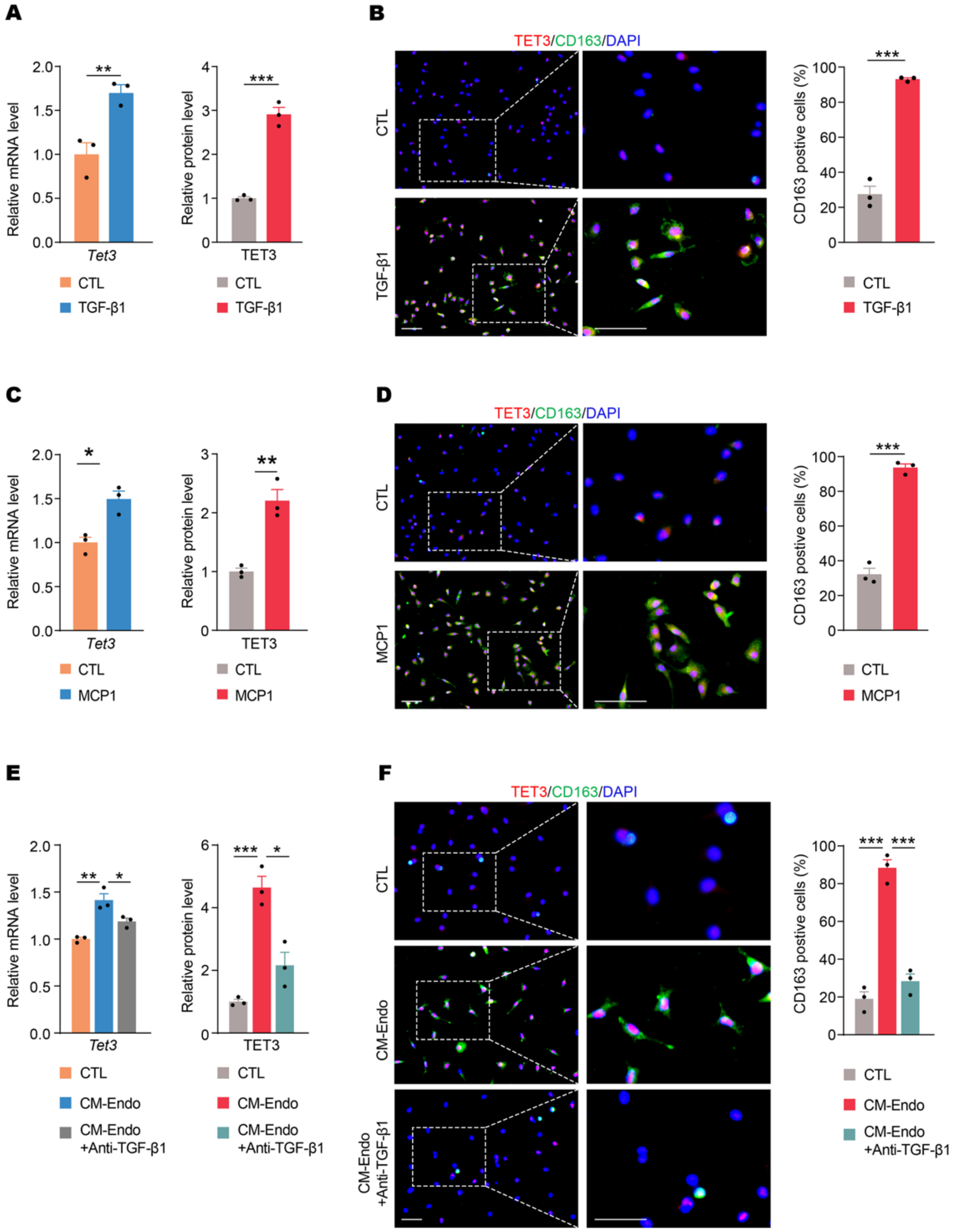

Figure S8. Macrophage TET3 expression is upregulated by inflammatory mediators in mouse peritoneal macrophages (PMs). (**A**) qRT-PCR of Tet3 mRNA (left) and IHC quantification of TET3 protein (right) from PMs isolated from WT mice treated with control media (CTL) or TGF-β1 at a final concentration of 30 ng/ml. RNAs and proteins were analyzed at 24 h and 48 h following TGF-β1 treatment, respectively. Immunostaining of TET3 (red) in **B** was used for quantification of TET3 protein expression. (**B**) Representative photomicrographs and corresponding statistical analysis of immunostaining of TET3 (red) and CD163 (green) in PMs treated as in **A**. (**C**) qRT-PCR of Tet3 mRNA (left) and IHC quantification of TET3 protein (right) from PMs treated with CTL or MCP1 at a final concentration of 200 ng/ml. RNAs and proteins were analyzed at 24 h and 48 h following MCP1 treatment, respectively. Immunostaining of TET3 (red) in **D** was used for quantification of TET3 protein expression. (**D**) Representative photomicrographs and corresponding statistical analysis of immunostaining of TET3 (red) and CD163 (green) in PMs treated as in **C**. (**E**) qRT-PCR of Tet3 mRNA (left) and IHC quantification of TET3 protein (right) from PMs treated with CTL, CM-Endo, or CM-Endo plus TGF-β1 antibody at a final concentration of 10 ng/ml for 72 h. MFI of TET3 in **F** was used to quantify TET3 protein expression. (**F**) Representative photomicrographs and corresponding statistical analysis of immunostaining of TET3 and CD163 in PM treated as in **E**. For quantification of immunostaining, n = 3 randomly selected areas per group were used. Data represent the mean ± SEM. *$P < 0.05$, **$P < 0.01$, and ***$P < 0.001$, by 2-tailed Student's *t* test. Scale bar: 40 μm.

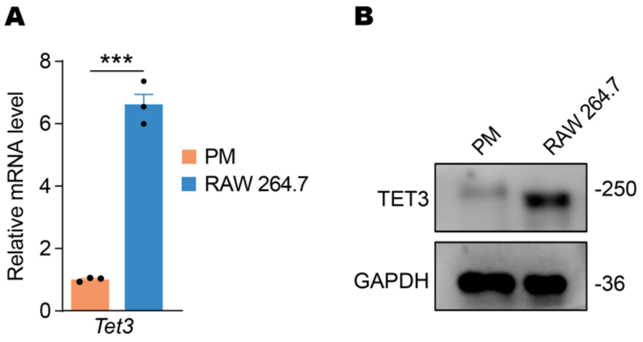

Figure S9. Unstimulated mouse RAW 264.7 cells overexpress TET3. (**A**) Relative Tet3 mRNA expression in mouse primary peritoneal macrophages (PM) and unstimulated RAW 264.7 cells. (**B**) Western blot analysis of proteins isolated from mouse PM and unstimulated RAW 264.7 cells. Note, the apparently slower mobility of the TET3 band in the PM was likely a result of posttranslational modification. Data represent the mean ± SEM. ****P* < 0.001, by 2-tailed Student's *t* test.

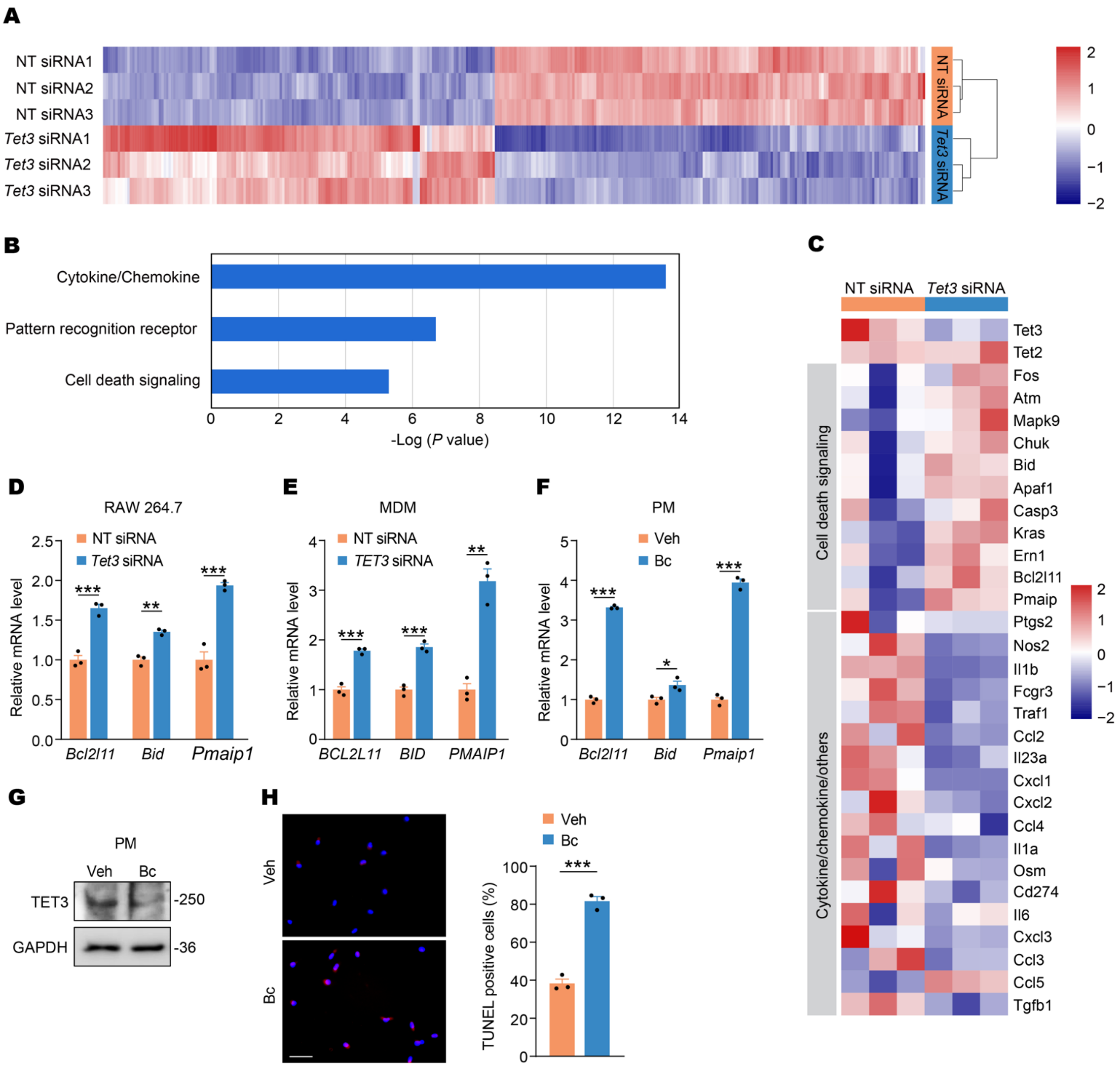

Figure S10. **TET3 affects the expression of inflammatory and apoptosis pathway genes.** (**A**) Unstimulated RAW 264.7 cells were transfected with NT siRNA or *Tet3* siRNA. RNAs were isolated 48 h later and subjected to genome-wide expression profiling by RNA-seq. Heat map of genes with significant expression changes ($p < 0.05$) are shown. Scale based on changes in log2 expression. $n = 3$ biological replicates in each group. (**B**) IPA of RNA-seq revealed 3 classes to be among the top affected by TET3 expression. (**C**) Heat map of selected genes up- or down-regulated in RAW 264.7 cells. (**D**) qRT-PCR of *Bcl2l11*, *Bid* and *Pmaip1* in unstimulated Raw 264.7 macrophages transfected with *Tet3* siRNA or NT siRNA for 48 h. (**E**) qRT-PCR of *BCL2L11*, *BID* and *PMAIP1* in human MDMs primed with TGF-β1 at 10 ng/ml and transfected with *TET3* siRNA or NT siRNA for 36 h. (**F**) Peritoneal macrophages (PM) were isolated from WT mice and treated with TGF-β1 at a final concentration of 30 ng/ml. After 48 h, vehicle or Bc was added at a final concentration of 10 μM and incubation carried out for 48 h. RNAs were extracted and analyzed by qRT-PCR. (**G**) Mouse PM were treated as in **F**. Proteins were isolated for western blot analysis. Representative immunoblots of TET3 are shown. (**H**) Mouse PM were treated as in **F**. TUNEL assays were performed after 48 h of treatment with Bc or vehicle. Representative photomicrographs and corresponding statistical analysis of TUNEL$^+$ (red) cells are shown. $n = 3$ randomly selected areas per group. All data represent the mean ± SEM. $^{**}P < 0.01$ and $^{***}P < 0.001$, by 2-tailed Student's t test. Scale bar: 40 μm.

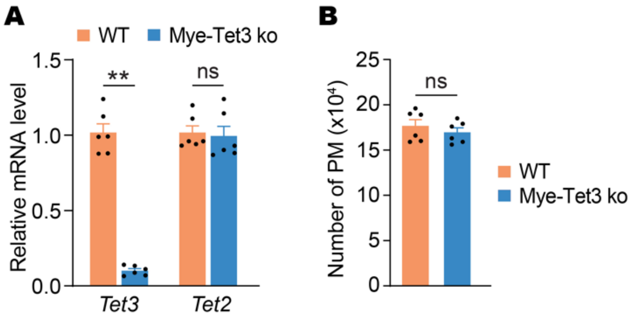

Figure S11. Mouse peritoneal macrophage numbers and gene expression. (**A**) qRT-PCR of Tet3 and Tet2 mRNAs showing ~90% decreased expression of *Tet3* (but not *Tet2*) in peritoneal macrophages of Mye-Tet3 ko mice compared to WT controls. (**B**) Peritoneal macrophage numbers of WT and Mye-Tet3 ko mice. n = 6 animals per genotype. Each dot represents an animal. Data represent the mean ± SEM. ** P < 0.01, by 2-tailed Student's t test. ns, not statistically significant.

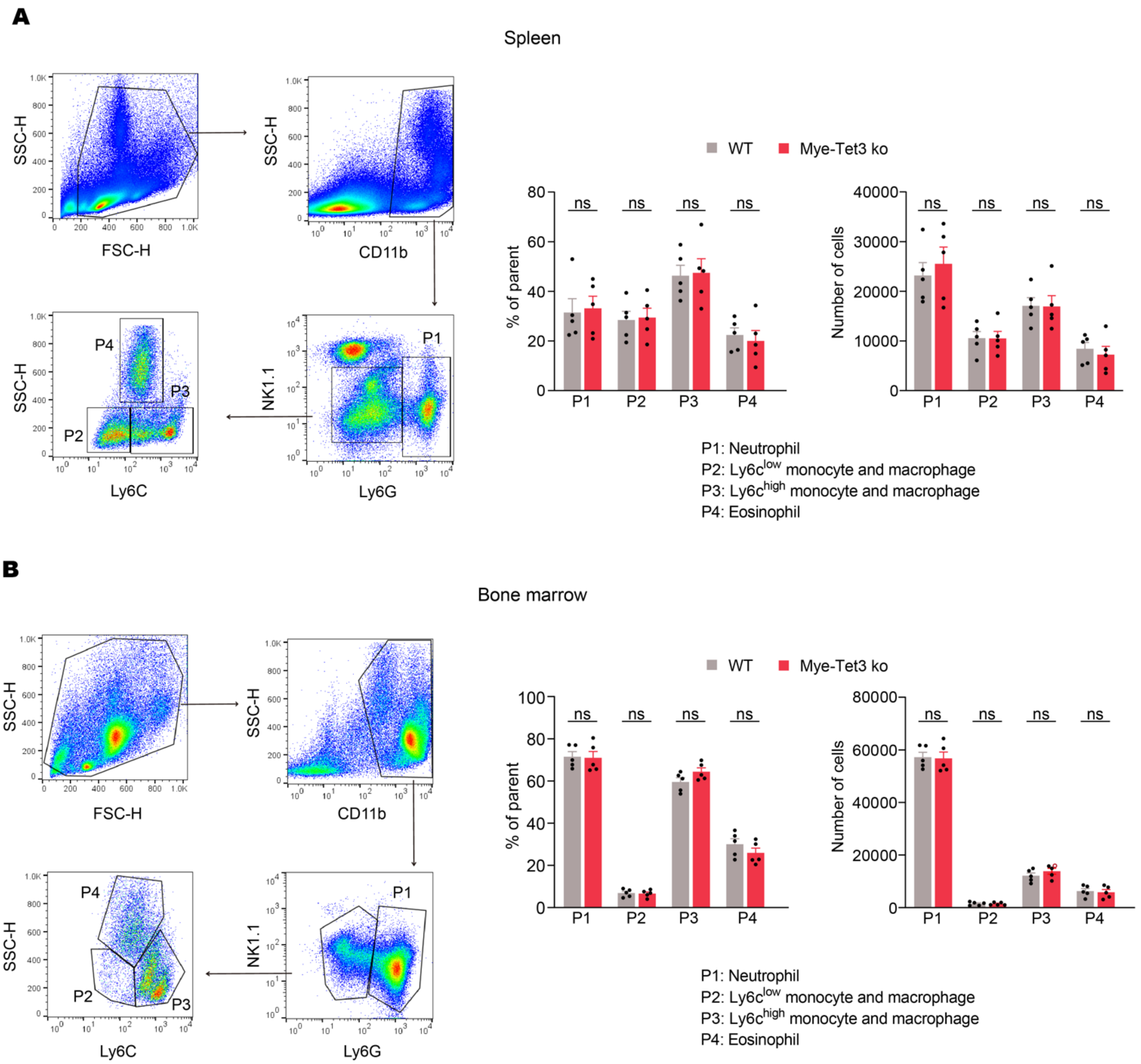

Figure S12. **Steady-state myeloid lineage characterization in WT and Mye-Tet3 ko mice.** (**A**) Myeloid lineage analysis of spleen. Splenocytes were harvested from WT or Mye-Tet3 ko mice. Cells were stained with the indicated markers. Left: representative flow cytometry plots and the gating strategy are shown. Right: quantification of the indicated populations, with values representing the population percentages within the parent gate and total numbers in spleen. n = 5 animals per group. (**B**) Similar analysis as in **A** was performed on bone marrow cells. *n* = 5 animals per group. Each dot represents an animal. All data represent the mean ± SEM. 2-tailed Student’s t test. ns, not statistically significant.

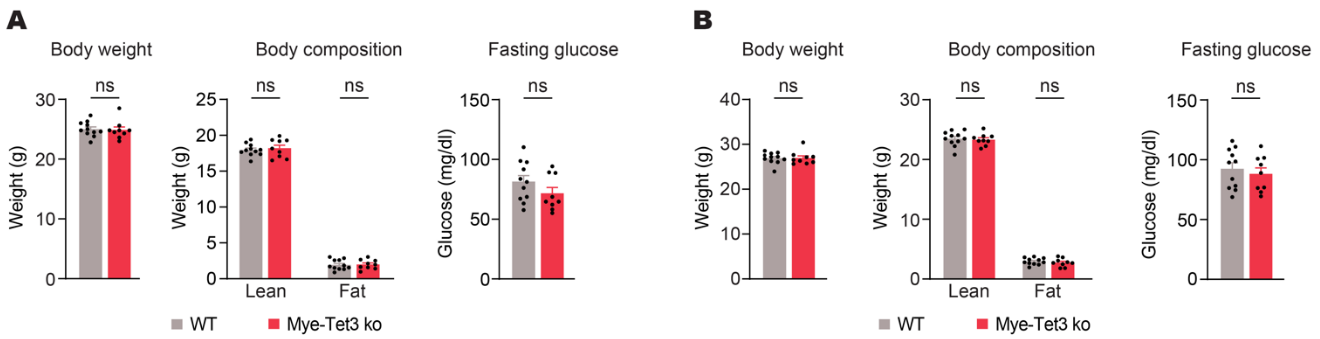

Figure S13. Body weight, body composition and fasting blood glucose levels of WT controls (*n* = 11) and Mye-Tet3 ko (*n* = 9) female mice at the age of 8 weeks (**A**) and 10 weeks (**B**), respectively. Each dot represents a mouse**.** Data represent the mean ± SEM. 2-tailed Student's t test. ns, not statistically significant.

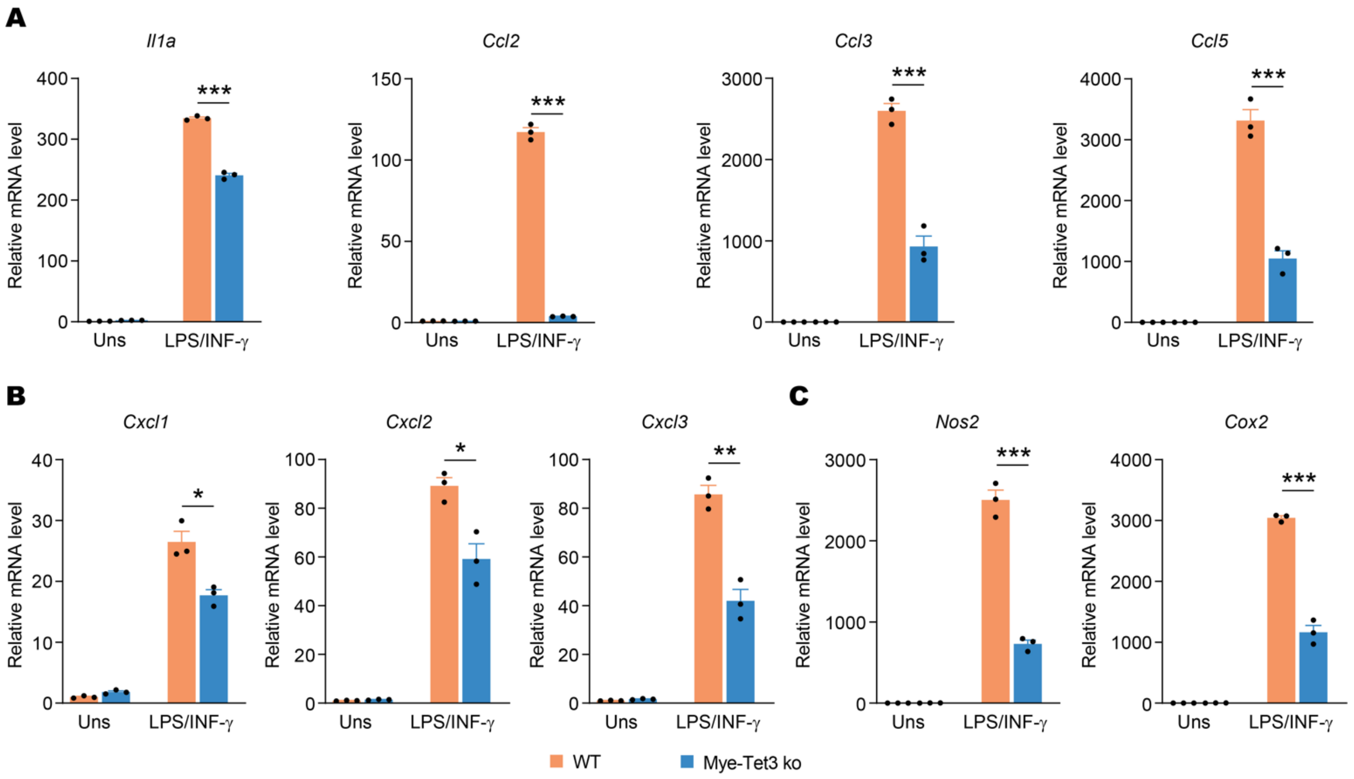

Figure S14. Effects of Tet3 deficiency on the expression of proinflammatory genes by cultured peritoneal macrophages. (**A**) Peritoneal macrophages were isolated from Mye-Tet3 ko mice or WT controls (*n* = 3 mice per genotype) and treated with 10 ng/ml LPS and 20 ng/ml IFN-γ. RNAs were isolated after 10 h (*Cxcl1*), 6 h (*Il1a*, *Ccl2*, *Ccl3*, *Ccl5*, *Cxcl2*, *Cxcl3*, *Cox2*), or 4 h (*Nos2*) of LPS/IFN-γ stimulation. mRNA levels of proinflammatory cytokines (**A**), chemokines (**B**) and enzymes (**C**) were quantified by qRT-PCR. All data represent the mean ± SEM. *P < 0.05, **P < 0.01, and ***P < 0.001, by 2-tailed Student's t test.

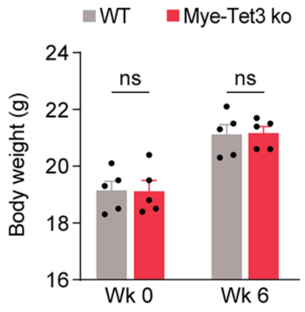

Figure S15. Body weight of mice. *n* = 5 animals per group. Each dot represents a mouse. Data represent the mean ± SEM. 2-tailed Student's t test. ns, not statistically significant.

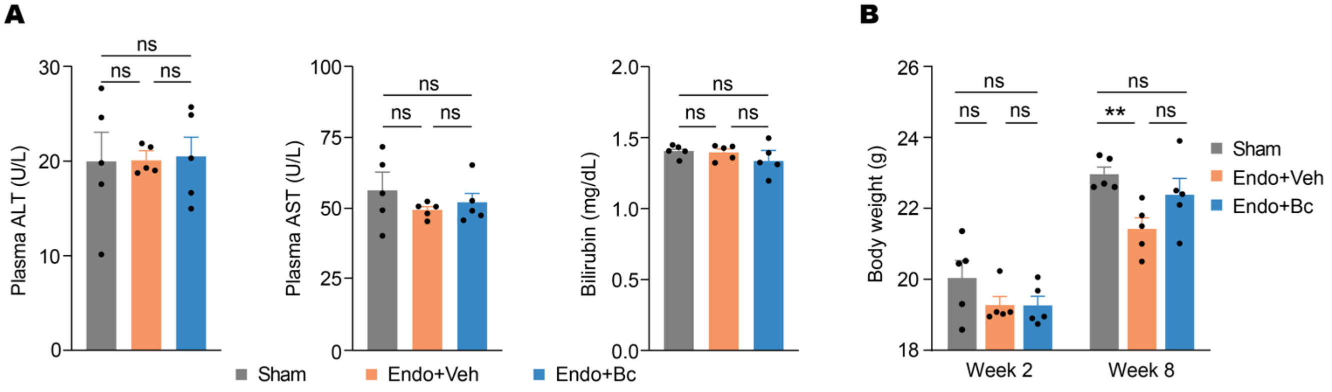

Figure S16. (**A**) Plasma alanine transaminase (ALT), aspartate transaminase (AST) and bilirubin from mice showing no differences between the groups. *n* = 5 animals per group. Each dot represents an animal. (**B**) Body weight of mice. *n* = 5 animals per group. Each dot represents an animal. All data represent the mean ± SEM. **P < 0.01, by 1-way ANOVA with Tukey's post-test. ns, not statistically significant.

A

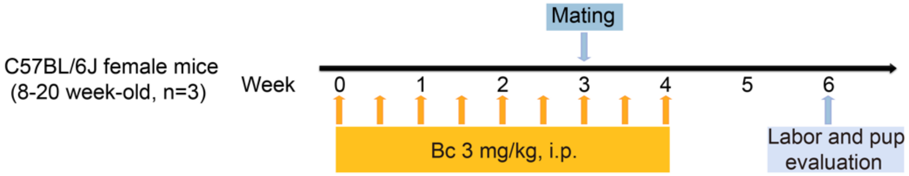

B

Mouse ID	Litter size
No.1	6
No.2	5
No.3	8

C

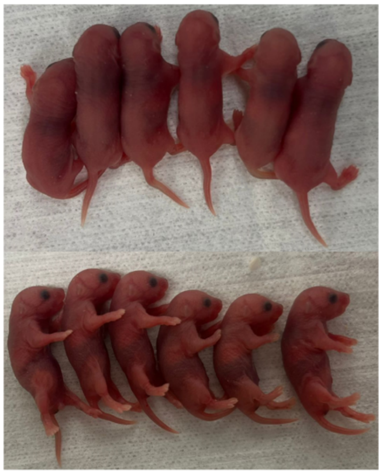

Figure S17. Bc does not affect fertility. (**A**) Schematic diagram of experiments. Female mice were i.p. injected with Bc at 3 mg/kg twice weekly for 3 weeks prior to mating. Mice were monitored weekly until delivery. All mice gave birth about 19-21 days after mating. (**B**) Litter sizes. (**C**) Image of pups from mouse No.1 showing no gross abnormality of the newborns.

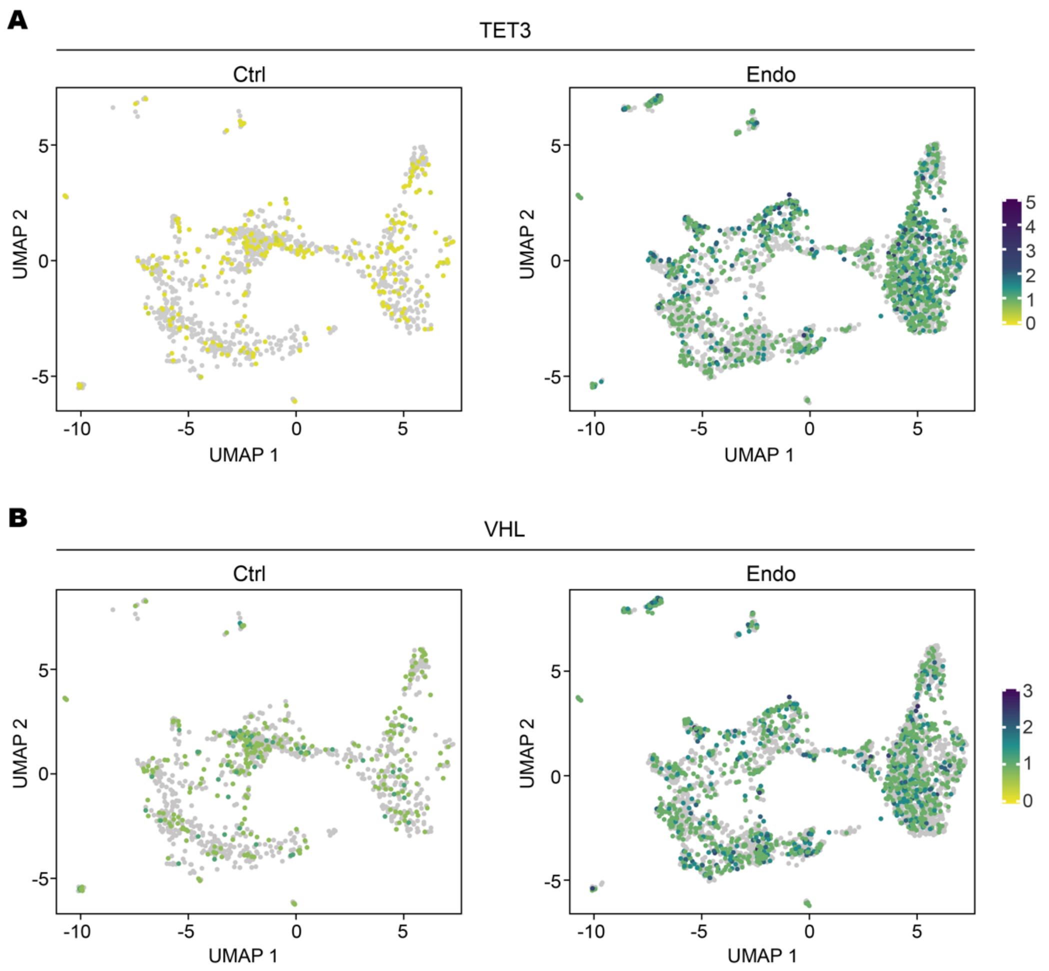

Figure S18. Human scRNA-seq macrophage UMAP showing TET3 OE macrophages (right panel, green dots) (**A**), with a large majority of which also overexpressing VHL (**B**, right panel, green dots) in Endo. *n* = 3 samples in each group.

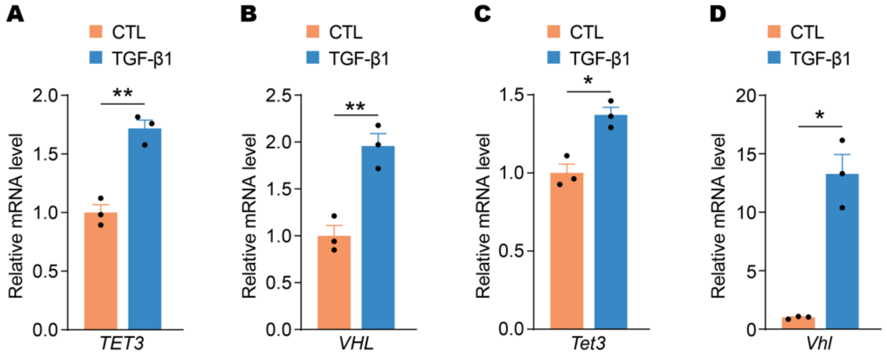

Figure S19. **TGF-β1 upregulates expression of both TET3 and VHL.** qRT-PCR of *TET3* mRNA (**A**) and *VHL* mRNA (**B**) from human MDMs treated with CTL or TGF-β1 at a final concentration of 10 ng/ml for 48 h. qRT-PCR of *Tet3* mRNA (**C**) and *Vhl* mRNA (**D**) from mouse PMs treated with CTL or TGF-β1 at a final concentration of 30 ng/ml for 24 h. Data represent the mean ± SEM. *P < 0.05 and **P < 0.01, by 2-tailed Student's t test.

Supplemental Table 1

Endometrium histology	Other drug treatment	Oral contraceptive treatment	rASRM Stage	Sample	Pregnancy	Ethnicity	Age	PID
Proliferative	-	-	0	Normal endometrium	1	White	35	Ctrl
Proliferative	-	-	0	Normal endometrium	0	Asian	35	Control #1
Proliferative	-	-	0	Normal endometrium	0	Asian	33	Control #2
Proliferative	-	-	0	Normal endometrium	1	Asian	37	Control #3
Proliferative	-	-	0	Normal endometrium	1	Asian	39	Control #4
Proliferative	Ibuprofen	-	3	Peritoneal endometriosis	1	White	33	Endo
Proliferative	-	-	4	Peritoneal endometriosis	2	Aisan	43	Patient #1
Proliferative	-	-	3	Peritoneal endometriosis	1	Asian	38	Patient #2
Proliferative	-	-	4	Peritoneal endometriosis	2	Aisan	35	Patient #3
Proliferative	-	-	3	Peritoneal endometriosis	2	Aisan	40	Patient #4
Proliferative	-	-	4	Peritoneal endometriosis	1	Asian	36	Patient #5
Proliferative	-	-	3	Peritoneal endometriosis	1	Aisan	38	Patient #6

Supplemental Table 2

Other drug treatment	Oral contraceptive treatment	rASRM Stage	Cohort	Pregnancy	Ethnicity	Age	GEO number	PID
Levothyroxine	Norethindrone/ E2:1/20	0	Control	1	Asian	36	GSM6102532	C01
Lexapro	Norethindrone/ E2:1/20	0	Control	0	White	22	GSM6102533	C02
Ibuprofen	-	0	Control	0	White	42	GSM6102534	C03
Liraglutide, Lisinipril, Januria	-	3	Endometriosis	0	White	40	GSM6595248	EP01
-	Norethindrone/ E2:1/20	2	Endometriosis	0	Hispanic	40	GSM6595250	EP02
Dostinex, Clonazepam, Lamictal, Seroquel, Sumatriptan	Norethindrone/ E2:1/20	4	Endometriosis	0	Hispanic	33	GSM6595252	EP03
Lexapro, Xanax, Klonopin	E2/ Drospirenone	4	Endometriosis	0	Asian	32	GSM6595261	EO01
-	Norethindrone	4	Endometriosis	2	Hispanic	35	GSM6102562	EO02
-	Norethindrone/ E2:1/20	4	Endometriosis	2	Hispanic	45	GSM6102556	EO03

Supplemental Table 3

qPCR primer sequences		
**Gene**	**Forward Primer**	**Reverse Primer**
Tet3 (mouse)	5'-TGCGATTGTGTCGAACAAATAGT-3'	5'-TCCATACCGATCCTCCATGAG-3'
Tet2 (mouse)	5'-AGCAAGAGATTCCGAAGGAT-3'	5'-AGTGGAGGACTGAGTGCAAG-3'
Il1b (mouse)	5'-CCCAACTGGTACATCAGCAC-3'	5'- TCTGCTCATTCACGAAAAGG-3'
Il6 (mouse)	5'-CTACCCCAATTTCCAATGCT-3'	5'- ACCACAGTGAGGAATGTCCA-3'
Cxcl1 (mouse)	5'-CCGAAGTCATAGCCACACTCAA-3'	5’-CAAGGGAGCTTCAGGGTCAA-3’
Cxcl2 (mouse)	5'-TGACTTCAAGAACATCCAGAGCTT-3'	5’-CTTGAGAGTGGCTATGACTTCTGTCT-3’
Cxcl3 (mouse)	5'-TTTGAGACCATCCAGAGCTTGA-3'	5’-CCTTGAGAGTGGCTATGACTTCTGT-3’
Ccl3 (mouse)	5'-GCGCCATATGGAGCTGACA-3'	5’-CGTGGAATCTTCCGGCTGTA-3’
Ccl5 (mouse)	5'-CAGCAGCAAGTGCTCCAATC-3’	5’-CACACACTTGGCGGTTCCTT-3'
Cox2 (mouse)	5'-TGGTGCCTGGTCTGATGA-3'	5’-GTGGTAACCGCTCAGGTGTTG-3'
Il1a (mouse)	5'-GCACCTTACACCTACCAGAGT-3'	5’-AAACTTCTGCCTGACGAGCTT-3'
Ccl2 (mouse)	5’-CAGCCAGATGCAGTTAACGC-3’	5’-GCCTACTCATTGGGATCATCTTG-3'
Nos2 (mouse)	5'-CACCTTGGAGTTCACCCAGT-3'	5’-ACCACTCGTACTTGGGATGC-3'
Bcl2l11 (mouse)	5'-GAGTTAGGGGCTGGCTCTAC-3'	5'- GACCAAGAAAGAGCACCTCA-3'
Bid (mouse)	5'-CTTGCTACGGAATGAAAGA-3'	5'- TTACCCAGGGAAAGAGGATG-3'
Pmaip1 (mouse)	5'-AGTCGTGGAGCTAGGGAAGT-3'	5'- ACATTGCTCTGCAGTTGTCA-3'
Lin28b (mouse)	5'-TTTGGCTGAGGAGGTAGACTGCAT-3'	5'-ATGGATCAGATGTGGACTGTGCGA-3'
Vhl (mouse)	5'-CTCAGCCCTACCCGATCTTAC-3'	5'-ACATTGAGGGATGGCACAAAC-3'
Rplp0 (mouse)	5'-GCTCCAAGCAGATGCAGCA-3'	5’-CCGGATGTGAGGCAGCAG-3'
BCL2L11 (human)	5'-TAAGTTCTGAGTGTGACCGAGA-3'	5'-GCTCTGTCTGTAGGGAGGTAGG-3'
PMAIP1 (human)	5'-ACCAAGCCGGATTTGCGATT-3'	5'-ACTTGCACTTGTTCCTCGTGG -3'
BID (human)	5'-ATGGACCGTAGCATCCCTCC-3'	5'-GTAGGTGCGTAGGTTCTGGT -3'
TET3 (human)	5'-GACGAGAACATCGGCGGCGT-3'	5'-GTGGCAGCGGTTGGGCTTCT-3'
TET2 (human)	5'-TTCGCAGAAGCAGCAGTGAAGAG-3'	5'-AGCCAGAGACAGCGGGATTCCTT-3'
IL1B (human)	5'-ATGATGGCTTATTACAGTGGCAA-3'	5'-GTCGGAGATTCGTAGCTGGA-3'
IL6 (human)	5'-CCTGAACCTTCCAAAGATGGC-3'	5'-TTCACCAGGCAAGTCTCCTCA-3'
VHL (human)	5'-GCAGGCGTCGAAGAGTACG-3'	5'-CGGACTGCGATTGCAGAAGA-3'
RPLP0 (human)	5'-GGCGACCTGGAAGTCCAACT-3'	5'-CCATCAGCACCACAGCCTTC-3'
