# Supplementary material for: TET3-overexpressing macrophages promote endometriosis
Source: J Clin Invest. 2024 Nov 1;134(21):e181839. doi: 10.1172/JCI181839 (PMC11527447; doi:10.1172/JCI181839)

## Figure 3B

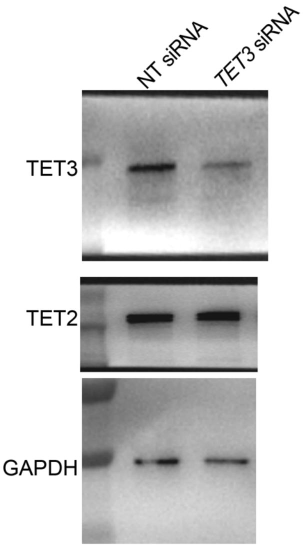


## Figure 3E

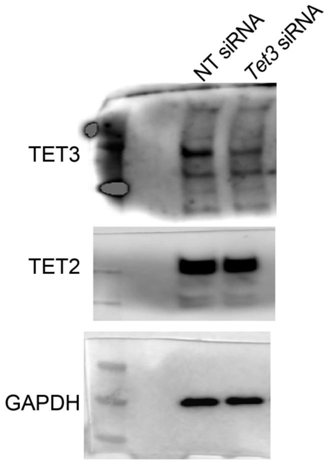


## Figure 4A

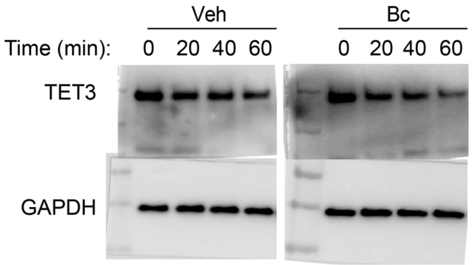


## Figure 4B

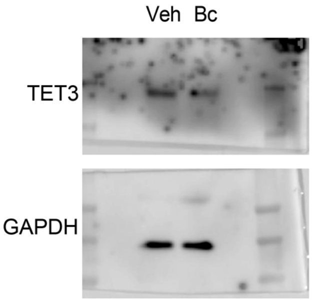


## Figure 4C

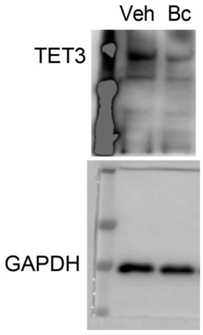


## Figure 4D

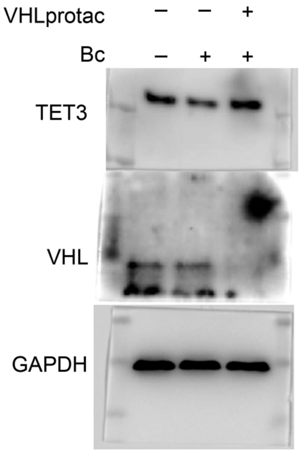


## Figure 4E

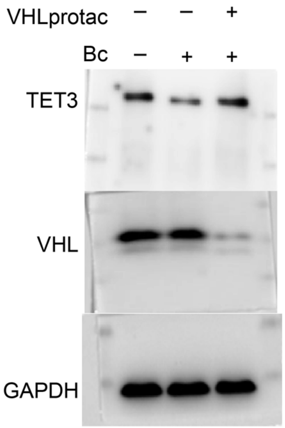


## Figure 4F

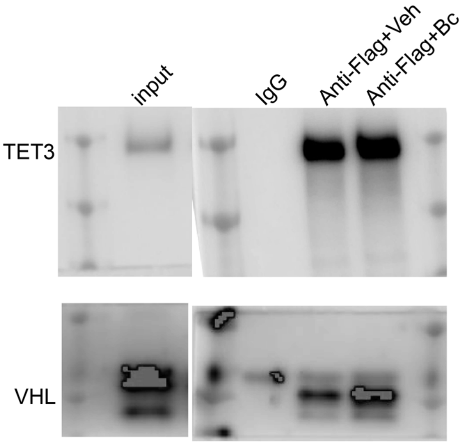


## Figure 4G

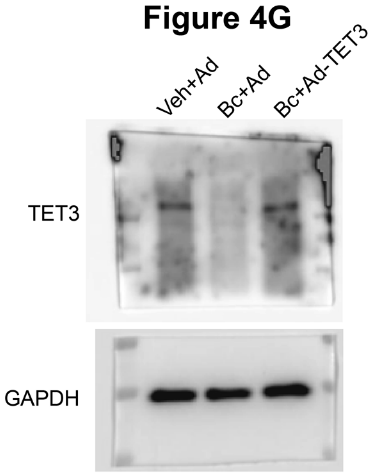


## Figure 4I

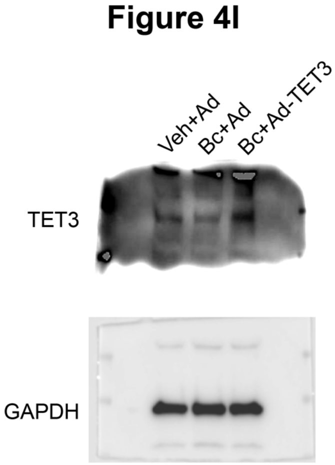


## Figure 5E

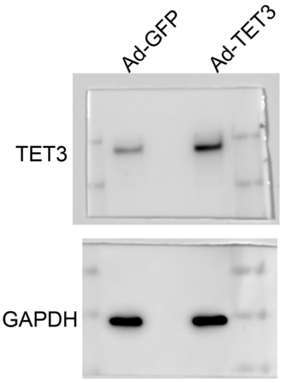


## Figure S9B

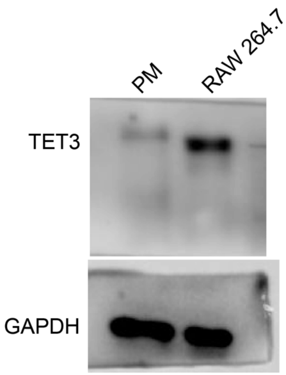


## Figure S10G

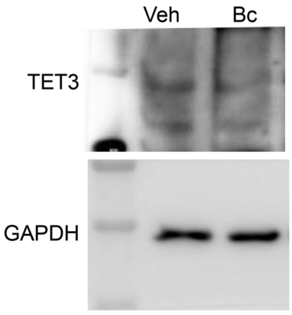

Supplement: Unedited blot and gel images [file jci-134-181839-s079.pdf]
